# Soil microbial and plant responses to increasing antibiotic concentration: a case study of five antibiotics

**DOI:** 10.1128/aem.01581-25

**Published:** 2026-02-24

**Authors:** Sarah van den Broek, Inna Nybom, Rafaela Feola Conz, Yifei Sun, Thomas D. Bucheli, Sebastian Doetterl, Martin Hartmann, Gina Garland

**Affiliations:** 1 Soil Resources, Department of Environmental Systems Science, Institute of Terrestrial Ecosystems, ETH Zürich 400186 , Zürich, Switzerland; 2 Sustainable Agroecosystems, Department of Environmental Systems Science, Institute of Agricultural Sciences, ETH Zürich 98840 , Zürich, Switzerland; 3 Environmental Analytics, Agroscope Reckenholz, Zürich, Switzerland; 4 Key Laboratory of Agro-Environment, Ministry of Agriculture, Institute of Environment and Sustainable Development in Agriculture, Chinese Academy of Agricultural Sciences 272684 , Beijing, China; 5 Soil Quality and Soil Use, Agroscope Reckenholz, Zürich, Switzerland; University of Georgia Center for Food Safety, Griffin, Georgia, USA

**Keywords:** antibiotics, soil microbial communities, antibiotic resistance, One Health

## Abstract

Antibiotic contamination from biogenic waste in agricultural soils poses a significant threat to soil health and crop productivity. We investigated the effect of antibiotics on the soil microbial community, antibiotic resistance genes, and mobile genetic elements (MGEs) and plant productivity in a 6-week greenhouse trial. Here, *Spinacia oleracea* (spinach) and *Raphanus sativus* (radish) were grown from seeds, and a mix of five antibiotics, namely sulfamethoxazole, trimethoprim, enrofloxacin, clarithromycin, and chlortetracycline, was added to the soil at concentrations of 0, 0.1, 1, and 10 mg kg^−1^ soil dry weight (c0, c0.1, c1, and c10, respectively). Overall, we found that the antibiotic treatments significantly impacted prokaryotic α-diversity and prokaryotic and fungal β-diversity. The relative abundance of human and plant pathogens did not increase under antibiotic exposure, but there was a significant reduction in plant growth-promoting bacteria. Moreover, the c10 treatment significantly increased the abundance of MGE *intI1,* indicative of horizontal gene transfer and sulfonamide resistance gene *sul1,* and significantly lowered radish biomass and nitrogen uptake, while spinach biomass and nitrogen uptake were unaffected. In summary, our study showed that antibiotic exposure significantly changed prokaryotic community diversity, while fungi remained largely unaffected. The reduction of plant growth-promoting bacteria may have a significant impact on soil nutrient cycling and crop productivity, but more research is needed to understand the long-term impact of these co-applied antibiotics on food production. Additionally, more studies are needed to understand the effect of antibiotics on realistic, field-scale conditions to fully understand the impact on environmental and human health.

**IMPORTANCE:**

Agricultural soils are frequently contaminated with complex mixtures of antibiotics from various biogenic sources, yet we lack a clear understanding of their specific ecological impact. While many studies investigate antibiotics, they are often studied in pollution sources like manure, which contain confounding factors like heavy metals. To provide a mechanistic understanding of antibiotic-specific responses, we investigated the effects of a complex, five-antibiotic mixture on the soil-plant system, independent of other contaminants. As expected, antibiotics reduced prokaryotic diversity and increased the abundance of some genes related to antibiotic resistance. Additionally, antibiotic exposure reduced plant growth-promoting bacteria, which may have subsequent detrimental effects on plant and soil health. Moreover, we found that antibiotic exposure can reduce plant biomass and nitrogen uptake, but this is highly plant dependent. This research highlights the critical need to monitor antibiotic pollution due to its potential detrimental effect on plant health and alterations to the soil microbiome.

## INTRODUCTION

There is a growing need for organic fertilizers to support not only food production but also environmental sustainability (1). It is increasingly recognized that recycling biogenic wastes like animal manure and human excreta into organic fertilizer is a way to increase fertilizer accessibility (2), soil health (3), and plant growth (4). However, these recycled organic fertilizers have also been shown to contain contaminants, such as antibiotics, microplastics, and heavy metals (5, 6). Due to the excessive and increasing use of antibiotics in human medicine and animal husbandry (7), antibiotics from animal manure and sewage sludge have been detected worldwide (8–10). Consequently, high concentrations of antibiotics end up in agricultural soils via fertilization with animal manure and sewage sludge (9) or through wastewater irrigation (11, 12). Antibiotics consist of complex molecules with varying functional groups and can be divided into several classes based either on chemical structure (13) or on the mode of action, such as inhibiting DNA replication or protein synthesis (14). Tetracycline antibiotics (inhibition of protein synthesis) are most frequently detected in animal manure and biosolids and generally among the highest detected concentrations of all classes of antibiotics. Other frequently detected antibiotic classes in organic fertilizers are fluoroquinolones (inhibition of DNA synthesis), sulfonamides (inhibition of folic acid synthesis), and macrolides (inhibition of protein synthesis) (8, 15, 16). The detected antibiotic concentrations generally remain below the 0.1 mg kg^−1^ range in agricultural soils worldwide, while occasionally higher concentrations have been reported, for example, 0.37 mg kg^−1^ fluoroquinolone in Austria and 0.3 mg kg^−1^ sulfonamide in the UK (8).

It has been found that once antibiotics enter the soil, they can decrease microbial diversity (17), change soil microbial community structure, and lower microbial biomass (18). In addition, while antibiotics can suppress certain plant pathogens in soil, such as *Pseudomonas fluorescens* and *Bacillus amyloliquefaciens* (19), they can also decrease the abundance of plant growth-promoting (PGP) microorganisms, such as *Streptomyces* and *Sulfuricaulis*, which are known to stimulate crop growth and improve crop health (20). Moreover, exposure to the sulfonamide sulfadiazine has been shown to increase microbial genera harboring pathogens like *Clostridium* and *Gemmatimonas* and lower bacterial genera associated with higher soil quality, such as *Lysobacter* and *Adhaeribacter* (21). As a result, there is growing concern that these changes may negatively affect soil nutrient cycling and crop health. Moreover, antibiotics can be directly phytotoxic for crops by damaging photosynthesis processes (22), reducing nutrient uptake (23), and causing oxidative stress (24), thereby delaying germination (23), reducing crop biomass (25), and lowering crop quality by reducing nitrogen (N) uptake (26).

In addition to impacts on soil and plant health, antibiotic exposure in agricultural systems following fertilizer application has been linked to increased presence of antibiotic-resistant bacteria (ARB) (9). While ARB occur naturally even in pristine environments due to resource competition among microorganisms (27), they are much more prevalent in environments with increased anthropogenic influence (28). ARB contain antibiotic resistance genes (ARGs) encoding the resistance mechanism to a specific antibiotic or a group of antibiotics, such as enzymatic activation proteins that break down the antibiotic or efflux pumps that transport the antibiotics outside the microbial cell. Mobile genetic elements (MGEs) can make ARGs mobile either within a genome or across bacterial species. ARGs associated with MGEs can then move through a population using horizontal gene transfer (HGT) (27), making MGEs an important driver of antibiotic resistance (29). Very high concentrations of antibiotics (≥10 mg kg^−1^) can be found in manure but are rarely detected in soil even when directly treated with manure (8, 15). Consequently, the concentration of antibiotics is often lower than the minimal inhibitory concentration (15, 27), which is the lowest chemical or pharmaceutical concentration that prevents the growth of bacteria or fungi. Bacteria may not be killed outright at these sub-inhibitory levels; instead, this low concentration can lead to increased mutation rates and enhanced HGT (30). Additionally, sub-inhibitory levels exert selective pressure, reducing the fitness of susceptible bacteria and favoring the proliferation of antibiotic-resistant strains. Conversely, high doses of antibiotics exert strong selective pressure, killing susceptible bacteria and inadvertently leaving an ecological niche open. This allows resistant bacteria to rapidly thrive and proliferate. This process has been extensively described in gut microbiome studies (31), and similarly, soil studies show that both low and high concentrations of antibiotics can increase ARGs (20, 32). The contribution of environmental ARGs to clinical events is difficult to determine due to technical constraints such as limited genomic surveillance (33, 34) but can have severe consequences for human health (27, 35). One way this transfer can happen is through consuming raw produce (36), and ARB and ARGs have been found on crops like radish (37) and spinach (38), which are often eaten raw. Therefore, the antibiotic exposure of soil microorganisms through recycled fertilizers and reclaimed water application is a potential risk for human health.

Considering the negative environmental consequences associated with antibiotic pollution, it is vital to understand how antibiotics affect the soil microbial community, as this may ultimately impact crop yields as well as environmental and human health. To date, research into the impact of antibiotics on the soil microbial community has largely focused on the direct impact of contamination sources (i.e., manure and sewage sludge). While these studies are important to understand real-life scenarios of environmental antibiotic dissemination, they often introduce not only antibiotics but also heavy metals and nutrients to the soil, thereby confounding the observed effects on both soil microbial communities and their antibiotic resistance. To better understand antibiotic-specific microbial responses with limited influence of complex environmental variables, mechanistic investigations under controlled conditions are necessary. Moreover, mechanistic studies often investigate at most three antibiotics at the same time (17, 18, 32), while agricultural soils can contain many antibiotics at the same time (9). The goal of this study, therefore, was to understand the impacts of five co-applied antibiotics on the soil prokaryotic and fungal community diversity and composition, ARGs and MGEs, and plant health. To simulate the exposure of antibiotics in organic fertilizers to the soil microbial community, we selected five structurally different antibiotics: the tetracycline chlortetracycline (CTC), the fluoroquinolone enrofloxacin (ENR), the macrolide clarithromycin (CLR), the sulfonamide sulfamethoxazole (SMX), and the diaminopyrimidine trimethoprim (TMP). SMX and TMP are commonly co-prescribed in both human and veterinary medicine and were both included due to their structural differences. Moreover, they represent the widely used anti-folate antibiotic group. These five antibiotics were co-applied to soil at varying concentrations of 0, 0.1, 1, and 10 mg kg^−1^ soil dry weight (DW) for each antibiotic equally. Their effects on soil microbial community structure, ARG and MGE absolute abundance, plant growth, and plant N uptake were studied in a greenhouse trial, where two crop species, spinach (*Spinacia oleracea*) and radish (*Raphanus sativus*), were grown to maturity for a total of 6 weeks. The effects of antibiotic addition to plant growth and nutrient uptake, soil microbial community diversity and taxa, ARGs, and MGEs were studied in soil samples after 6 weeks of exposure. We hypothesized that the increasing antibiotic concentrations would change the soil-microbe-plant system by (i) reducing microbial α-diversity and altering microbial β-diversity, with a stronger effect on the prokaryotic community compared to the fungal community, (ii) changing the microbial taxonomic composition, leading to reduction of PGP microorganisms, while increasing antibiotic-resistant microorganisms, (iii) increasing ARGs and MGEs, and (iv) decreasing plant productivity.

## MATERIALS AND METHODS

### Greenhouse trial and sample collection

Swiss topsoil (0–20 cm), free from the selected antibiotics and with a pH of 7.4, total organic carbon of 2.6%, and total N of 0.23%, was used in the experiment. Further details on the soil properties are provided in Table S1. The soil was dry sieved to Ø 5 mm prior to use. Antibiotics used in this study were chosen to represent a range of physicochemical characteristics, for example, with molecular weight from 253 (SMX) to 748 g mol^−1^ (CLR), water solubilities from 1.7 (CLR) to >600 mg L^−1^ (SMX, CTC), and consisting of different functional groups (15, 39–46). Further details on the chemicals used for the antibiotic analysis and physicochemical characteristics of the antibiotics are provided in Tables S2 and S3. Antibiotics were added to the soil to reach nominal individual antibiotic concentrations of 0.1, 1, and 10 mg kg^−1^ soil DW (further referred to as c0.1, c1, and c10). The stock solutions were prepared individually in methanol (TMP, CTC, and ENR) or acetone (SMX and CLR) and added to autoclaved sand (500 g). A corresponding volume of pure solvents was added to the control units without antibiotic addition (namely 4.5 mL of MeOH and 2 mL acetone) (antibiotic concentration 0 mg kg^−1^, further referred to as c0). The nominal and measured antibiotic concentrations are shown in Table S4. The sand was mixed thoroughly by shaking in a plastic bag. The bags were left open for 30 min after spiking to allow the solvents to evaporate, and then, pre-weighed soil (950 g) was added to the sand and mixed thoroughly. Sand amendment was used to ensure homogeneous antibiotic mixing and inhibit soil compaction during the experiment (soil:sand ratio: 3:1 [vol:vol]). To verify that the spiking was successful and confirm the antibiotic concentrations at D0, additional pots for each treatment were prepared (*n* = 6). The additional pots were spiked and prepared accordingly, sampled directly (D0) by collecting 150 g of the soil-sand mixture, and stored at −20°C until analysis.

To establish the experimental replicates, the spiked soil-sand mixture was transferred to pots (Ø 15 cm), and three pre-germinated spinach (*S. oleracea*) (48 h, 20°C, in dark) or radish (*R. sativus*) (12 h, 20°C, in dark) seeds were planted in each pot, with six replicates of each antibiotic concentration and plant treatment combination. Pots were watered to 80% water holding capacity and irrigated every second day throughout the experiment. A greenhouse trial was conducted at controlled climatic conditions (set temperature 20°C, range 19°C–24°C, 16:8 light:dark), and the duration of the experiment was set to 6 weeks. The experiment followed a randomized complete block design (47), where experimental units were divided into six blocks, each containing one replicate of each treatment. The experiment was established over the course of three consecutive days, where two blocks were started each day. The positions of the pots within the blocks and the block’s position in the greenhouse were randomized weekly. After 6 weeks (D42), the plants were harvested, the aboveground and belowground biomass were weighed separately, and the length of roots and shoots was measured. The bulk sand-soil mixture was gently homogenized prior to sample collection. Two separate bulk sand-soil mixture samples were collected: 50 g for microbial analyses and 300 g for the determination of soil total antibiotic concentrations and soil pH. The plant and soil samples were stored at −20°C immediately after collection until further processing.

### Antibiotic analysis

The total antibiotic concentration of the bulk sand-soil mixture was determined following the method described by Shi et al. (48), with the exception that the extract cleanup with dispersive solid-phase extraction was not conducted, as it was found not to be required after preliminary testing (results not shown). The acidity-regulated extraction-partition-concentration protocol by Shi et al. (48) is based on sample extraction with solvents (acetonitrile, acidified with 5% formic acid and potassium phosphate buffer, pH 3). The extraction method and chemicals used in the extraction are described in further detail in the supplemental material (Text S1). The bulk sand-soil samples were extracted directly after thawing. Here, approximately 2.5 g (c0, c0.1, and c1) or 1.25 g (c10) of moist sample was extracted. The determined total concentrations were calculated based on exact sample masses and corrected with pre-determined DW content of the sample (80.5% ± 1.6%) and soil content in the sand-soil mixture (65% DW). Extracted samples were analyzed on an Agilent liquid chromatography-triple quadrupole mass spectrometry system (LC-MS/MS, 6470, Agilent Technologies). The absolute recoveries ranged from 77.5% (SMX) to 104.8% (CLR), and the determined limits of quantification (LOQs) were ≤3.19 µg kg^−1^ DW, with the highest LOQ observed with CTC (Table S5). Further details on analytical method, and instrument and method precision are provided in the supplemental material (Text S2 and S3).

### Plant nutrient and soil pH analysis

A subsample of the frozen radish roots (belowground) and leaves (aboveground), as well as spinach leaves (not enough material collected for spinach root assessment), was dried (40°C, 7 days), ground into a fine powder, homogenized, and subsampled to measure nitrogen (N) and water content. The biomass of spinach roots was too small to sample for analysis, and therefore, only the spinach leaf (aboveground) samples were processed. Total moisture content for each plant was determined gravimetrically and used to calculate the DW biomass for each plant. The dried plant material was used to assess the total C and N content via dry combustion (LECO CHN628 Series Elemental Determinator) with isotope ratio mass spectrometry (IRMS) using the Elemental Analyzer Thermocycler Flash IRMS (EA IsoLink CN) and IRMS Delta V Plus Isotope Ratio MS (Thermo Fisher Scientific, Switzerland). Two different plant standards (124 *Medicago sativum* [44.8% C and 2.8% N] and 172 *Prunus laurocerasus* [48.0% C and 1.2% N], International Plant-Analytical Exchange, Wageningen Evaluation Programs for Analytical Laboratories) were included in the analysis to ensure the validity of the results. In case the sample mass of individual replicates was too small for sample analysis, the missing values of C and N in roots (radish only) and leaves (radish and spinach) were filled using mean replicate values (radish roots: c1 one missing value, c10 four missing values; radish leaves: c1 one missing value, c10 three missing values; and spinach leaves: c1 one missing value, c10 one missing value). Soil pH was determined in a 0.01 M CaCl_2_ solution using a pH meter (713 pH Meter, Metrohm, Switzerland).

### DNA extraction and sequencing analysis

For metabarcoding, DNA extraction of sand-soil samples (250 ± 2 mg) was performed in randomized order with the DNeasy PowerSoil Pro Kit according to the manufacturer’s instructions using the QIACube Connect System (Qiagen, Hilden, Germany). PCR was conducted on the normalized samples targeting the 16S rRNA gene (V4 region) with 341F and 806R primers (49) and the ribosomal ITS region with ITS3ngs and ITS4ngs primers (50) (Microsynth, Balgach, Switzerland) using TRUESEQ sequencing tags (Illumina, San Diego, CA, USA) (see Table S6 for the primer sequences and Text S5 for more details on the PCR protocol). PCR amplification was conducted using three technical replicates. The triplicate PCR products were pooled and sent to the Functional Genomics Center Zurich (FGCZ, Zurich, Switzerland). For indexing, a second PCR was performed by mixing 0.3 µM of NEXTERA containing Illumina’s unique dual indexes (IDT, Leuven, Belgium) with 2 µL of the first PCR pooled product and 1× GoTaq Colorless Master Mix, following the protocol for amplification (initial denaturation at 95°C for 3 min; 8 cycles of denaturation at 95°C for 40 s, annealing at 55°C for 15 s, elongation at 72°C for 2 min, and final elongation at 72°C for 5 min). Products were purified using Sera-Mag Select Beads (GE Cytiva Europa GmbH), quantified with the TapeStation 4200 electrophoresis system (Agilent Technologies, Waldbronn, Germany) pooled in equimolar ratios before preliminary sequencing on the Illumina NextSeq 2000 platform, spiking with PhiX at 30% (Illumina) to inform library re-pooling to better align read counts across samples. Final sequencing was conducted using the v3 chemistry (PE300) on the Illumina NextSeq 2000 platform (Illumina), spiking with PhiX at 30% (Illumina).

### Analyses of soil prokaryotic and fungal communities

A customized bioinformatics pipeline was used to process the sequencing data as previously described (51). Briefly, the sequence data quality was inspected using FastQC version 0.11.9 (52) and VSEARCH version 2.21.2 (53). PhiX was removed using Bowtie2 version 2.4.5 (54), primers were trimmed using cutadapt version 5.1 (55), and polyG tails were removed with fastp version 0.23.2 (56). Forward and reverse reads were merged (minimum merge length 300, quality truncated at phred score 7) using the fastq_mergepairs function in VSEARCH, and low-quality reads (expected error > 1) were filtered using the fastq_filter function in VSEARCH. Filtered reads were dereplicated, delineated, and chimeras were removed using the functions derep_fulllength, cluster_unoise, which uses the UNOISE algorithm (57), and uchime3_denovo, which uses the UCHIME algorithm (58) in VSEARCH. Metaxa2 version 2.2.3 (16S rRNA gene) (59) and ITSx version 1.1.3 (ITS2 region) (60) were used to verify the target region. SILVA version 138.1 (61) and UNITE version 9.0 (62) databases were trimmed to match the target region spanned by the primers using cutadapt, and taxonomy was assigned using the sintax algorithm (63) in VSEARCH using a cutoff of 70%.

### Database screening for pathogens and plant-beneficial microorganisms

Relevant pathogens and plant-beneficial bacteria were identified in our samples by comparing the taxonomic classifications to multiple databases. The prokaryotic genera and species were screened against the databases using the dplyr R package version 1.1.4 (64) using RStudio version 2024.04.2+764 (65), with R version 4.4.1 (66). Bacterial human pathogens were identified at the species level using a list of 1,513 infectious bacterial pathogens compiled by Bartlett et al. (67). Bacterial plant pathogens and plant-beneficial bacteria were identified at genus level using the plant-beneficial bacteria (PBB) database, containing 398 genera, and the Phytopathogen database, containing 258 species, collected by Li et al. (24). The database by Li et al. (68) further specifies the PBB into broad categories such as biocontrol and stress resistance, and subcategories such as N fixation and siderophore production. Fungal pathogens (animal or plant pathogen) and PGP fungi (endophytic, epiphytic, and saprotrophic fungi) were identified using FUNGuild and the authors’ recommended protocol (69). For the FUNGuild database categorization, pathogens and parasites were classified as animal or plant pathogenic, while ectomycorrhizal, endophytic, epiphytic, arbuscular mycorrhizal, ericoid, and saprotrophic fungi were classified as plant-beneficial. When a genus was identified as both beneficial and pathogenic according to the PBB database, it was classified based on the most likely scenario according to the literature. To supplement the genera with potential phenotypic and metabolic capacities related to antibiotic degradation, production, and resistance, the genera significantly impacted by antibiotic treatment were screened for common ARGs in the Comprehensive Antibiotic Resistance Database (CARD) (70) and antibiotic-related mechanisms using Web of Science using the keywords “genus” AND (“pollutant degradation” OR “antibiotic degradation” OR “antibiotic production” OR “antibiotic resistance”). This allowed for the classification of genera beyond pathogenic and PGP capacities. Based on the CARD results and the literature search, the genera were categorized as “pollutant degrader,” “antibiotic degrader,” “antibiotic producer,” “antibiotic resistant,” “ARG carrier,” or “unclassified.” Genera associated with ARGs but not confirmed carriers were categorized as unclassified. The results were published on Figshare (71).

### PCR and quantitative PCR of ARGs and MGEs

ARGs that are commonly associated with the application of organic fertilizers and are related to the resistance to the selected antibiotics were chosen for PCR and qPCR analysis, namely *sul1* (SMX resistance) (72, 73), *dfrA12* (TMP resistance) (74), *tetQ* (CTC resistance) (75), and *qnrS1* (ENR resistance) (76). CLR resistance was not measured because this requires a detailed investigation of the 23S gene, which is different across prokaryotic genera (77), and this was outside the scope of this research. MGEs *intI1* and *intI2* were chosen because of their associated resistance to sulfonamides and TMP, respectively (78, 79). For qualitative detection of the selected ARGs and MGEs via PCR, six primer sets targeting four ARGs and two MGEs were selected based on the literature (80–84) (see Table S6 for primer sequences). The validity of the selected primers was confirmed with *in silico* PCR. All selected genes were downloaded from the NCBI Nucleotide database on 04 April 2023 (85), and matches to the primer were tested using cutadapt and analyzed in RStudio using the seqinr R package version 4.2-36 (86). Then, PCR and qPCR were conducted (see Text S6 for more details).

### Statistical analyses

All statistical analyses were conducted in R version 4.4.1 (66), using RStudio version 2024.04.2+764 (65). Visualizations were made using R packages ggplot2 version 3.5.2 (87), patchwork version 1.3.0 (88), and RColorBrewer version 1.1-3 (89) and refined in Adobe Illustrator (90). Statistical analysis of metadata (soil pH, plant biomass, plant N, and antibiotic concentration [D42]) was conducted by checking for normality of data distribution using the Shapiro-Wilk test from the stats R package version 4.4.1 (66), normality of residuals using the Levene’s test from the car R package version 3.1-3 (91), and heteroscedasticity with the Breusch-Pagan Test from the lmtest R package version 0.9-40 (92). An ANOVA was conducted, followed by a TukeyHSD from the stats R package when the data and residuals were normally distributed, and no heteroscedasticity was found. A Kruskal-Wallis test from the stats R package was used, followed by a pairwise comparison using the Dunn Test from the rstatix R package version 0.7.3 (93) with Benjamini-Hochberg false discovery rate (FDR) multiple testing correction if the data were not normally distributed. Significance letters based on the statistical tests were determined with the multcompView R package version 0.1-10 (94).

Quality control of the sequencing data was ensured by investigating read quality (phred scores), read length distribution, and sequencing depth. Changes in α-diversity (observed richness, Pielou’s evenness, and Shannon diversity) and β-diversity (Bray-Curtis dissimilarity) were calculated from 100-fold iteratively subsampled amplicon sequence variant (ASV) count tables to account for differences in sequencing depth (95, 96). A permutational analysis of variance (PERMANOVA) using the vegan R package version 2.6-8 (97), followed by a pairwise comparison using the pairwiseAdonis R package version 0.4.1 (98), was conducted to determine significant differences between the β-diversity (Bray-Curtis dissimilarity) indices. For β-diversity, a principal coordinates analysis using the stats R package was conducted (cmdscale function). The vegan R package was used to determine significant effects of plant type and metadata (soil pH, plant biomass, plant C, plant N, antibiotic concentration [D42], ARGs, and MGEs) on β-diversity. First, a full distance-based redundancy analysis (partial dbRDA) model was created using plant type and all metadata as response variables with the capscale function. We did not include treatment in the model here because it would obscure the significant influence of the individual antibiotic concentrations at D42. Then, the significance of each variable within this full model was assessed using the permutest function. The significant variables were then used to make the partial dbRDA model using the capscale function, namely plant biomass, *sul1* gene copies normalized to *16S* rRNA gene copies (further referred to as *sul1 16S*^−1^ abundance), CTC concentration (D42), ENR concentration (D42) for prokaryotes and plant type, plant biomass, *sul1 16S*^−1^ abundance, and leaf N concentration for fungi. The first two constrained axes of the partial dbRDA model were visualized using an ordination plot. When assessing soil antibiotic concentrations, values below LOQ were replaced with 0. To assess the influence of antibiotic treatment, plant type, and their interaction on the abundance of prokaryotic and fungal taxa, individual PERMANOVA was conducted for each phylum and genus using Euclidean distance. The models included antibiotic concentration, plant type, and their interaction, and the results from these analyses were corrected for multiple testing using FDR multiple testing correction with the p.adjust function from the stats R package. The results were categorized as significantly impacted by antibiotic treatment when *P ≤* 0.05.

To further classify microbial genera significantly impacted by antibiotic treatment, the relative abundance on the genus level was assessed using generalized linear models. Relative abundances were modeled using a quasibinomial family with a logit link function, a common choice for proportional data that often exhibits overdispersion. A small constant (1 × 10^−7^) was added to all relative abundance values to handle zero observations. A linear model was fitted for each genus-plant combination (relative abundance ∼ concentration), and the statistical significance of the linear term was evaluated using Type II Wald χ2 tests from the car R package. Based on these tests, genera were categorized into three response groups: (i) monotonic increase: assigned when the linear term for concentration was statistically significant (*P <* 0.05) and positive, (ii) monotonic decrease: assigned when the linear term for concentration was statistically significant (*P <* 0.05) and negative, and (iii) non-monotonic: assigned when the linear term was not significant despite the genus being identified as significantly impacted by the antibiotic treatment in the initial analysis. To determine significant interactions between the ARGs and MGEs found with qPCR (ARGs and MGEs) and the soil parameters (soil pH and soil antibiotic concentrations at D42) for both plants, Spearman correlation analysis was conducted with the rcorr function of the Hmisc R package version 5.2-4 (99) and corrected for multiple testing with the stats R package. Spearman’s rho was set to 0 for non-significant correlations (*P ≤* 0.05), and the correlations were visualized using the corrplot R package version 0.95 (100).

## RESULTS

### Soil antibiotic concentration dynamics

To verify dosing accuracy, antibiotics of the stock solution were extracted and analyzed with LC-MS/MS. This demonstrated that the concentrations were in line with the intended spike levels, except for the antibiotic CLR 0.1 mg kg^−1^ treatment spike stock. For CLR, an order of magnitude lower concentration was detected due to an error in stock solution preparation, which was noted too late for correction (Text S4; Table S4). The lower spike concentration of CLR was consequently also reflected in the determined soil concentrations at D0 and D42 in the range of 0.013–0.014 mg kg^−1^ from c0.1 treatment samples (Table 1). The D0 soil concentrations of ENR, SMX, and CTC were lower than expected based on the spiked concentrations (Table S4). For example, the determined concentrations in c10 treatment soil were 8.4, 6.8, and 5.2 mg kg^−1^, respectively. Over the course of the experiment, the concentrations of CTC, SMX, and TMP decreased significantly; for example, for SMX, the measured soil concentrations at D42 were ≤16% of the measured concentrations at D0 samples (Table 1). On the contrary, the concentrations of ENR and CLR remained largely stable, excluding the lowest ENR treatment (c0.1), where the observed soil concentration declined from 0.06 mg kg^−1^ at D0 to 0.04 mg kg^−1^ at D42.

**TABLE 1:** Soil antibiotic concentrations in the different antibiotic treatments at the start (D0) and end of the trial (D42)^*a*^

Antibiotic treatment	Measured start (D0) (mg kg^−1^)	Measured end (D42) Radish (mg kg^−1^)	Measured end (D42) Spinach (mg kg^−1^)
Chlortetracycline (CTC)			
c0	N.D.	N.D.	N.D.
c0.1	0.089 ± 0.02	0.027 ± 0.01***	0.037 ± 0.001***
c1	0.95 ± 0.28	0.27 ± 0.02***	0.24 ± 0.01***
c10	5.28 ± 1.02	1.95 ± 1.33***	2.02 ± 0.08***
Clarithromycin (CLR)			
c0	N.D.	N.D.	N.D.
c0.1	0.014 ± 0.002	0.014 ± 0.001	0.013 ± 0.001
c1	1.09 ± 0.25	1.16 ± 0.05	1.06 ± 0.10
c10	11.33 ± 2.82	11.17 ± 0.94	11.23 ± 0.59
Enrofloxacin (ENR)			
c0	N.D.	N.D.	N.D.
c0.1	0.062 ± 0.006	0.037 ± 0.002***	0.037 ± 0.002***
c1	0.58 ± 0.15	0.53 ± 0.05	0.53 ± 0.03
c10	8.43 ± 1.57	8.86 ± 0.86	9.01 ± 0.66
Sulfamethoxazole (SMX)			
c0	N.D.	N.D.	N.D.
c0.1	0.041 ± 0.005	0.007 ± 0.001***	0.005 ± 0.001***
c1	0.49 ± 0.11	0.074 ± 0.02***	0.067 ± 0.02***
c10	6.79 ± 1.36	1.07 ± 0.21***	1.092 ± 0.09***
Trimethoprim (TMP)			
c0	N.D.	N.D.	N.D.
c0.1	0.13 ± 0.02	0.004 ± 0.0004***	0.004 ± 0.001***
c1	1.12 ± 0.22	0.043 ± 0.004***	0.041 ± 0.006***
c10	10.79 ± 2.20	4.82 ± 0.47***	4.82 ± 0.37***

^*a*^ Significance between measured start (D0) and measured end (D42) concentrations was determined with Kruskal-Wallis, *P ≤ 0.05, **P ≤ 0.01, and ***P ≤ 0.005. N.D., not detected.

### Impact of antibiotics on the soil microbial community

**Fig 1:**
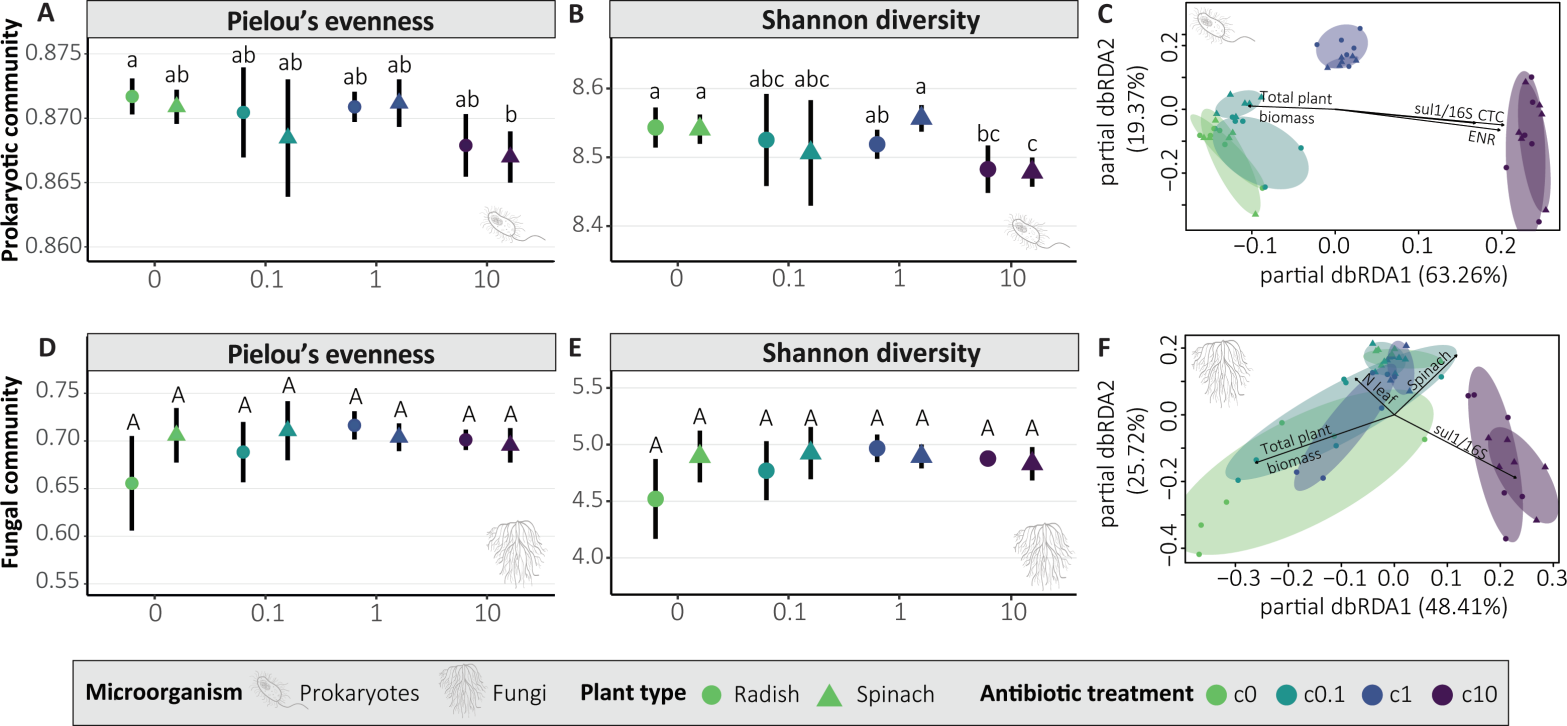
α- and β-diversity of soil prokaryotic and fungal communities. Differences in lowercase letters and uppercase letters indicate significant differences between plant type and antibiotic treatments for prokaryotic and fungal communities, respectively (*P* ≤ 0.05, see also Table S7) (**A, B, D, and E**). Distance-based redundancy analysis of prokaryotic (**C**) and fungal (**F**) communities. Arrows show which plant and soil properties significantly impacted β-diversity (*P* ≤ 0.05) (**C and F**). CTC, chlortetracycline and ENR, enrofloxacin (**C and F**).

In total, we found 37,089 prokaryotic ASVs and 3,605 fungal ASVs. Prokaryotic α-diversity was significantly influenced by antibiotic treatments and plant type as determined by pairwise PERMANOVA. Observed richness, Pielou’s evenness, Shannon diversity index, and inverse Simpson diversity index decreased with increasing antibiotic concentration (Fig. 1A and B; Table S7). For the fungal community, there were no significant effects for both antibiotic treatment and plant type (Fig. 1D; Table S7). Observed richness, Shannon diversity, and inverse Simpson were much higher for prokaryotic communities than for fungal communities (Table S7). Prokaryotic β-diversity was significantly affected by antibiotic treatment (*P =* 0.001) but not plant type (*P =* 0.457) as determined by PERMANOVA. Pairwise comparisons (FDR-adjusted) showed that β-diversity did not significantly differ between the c0 and c0.1 treatments (*P =* 1.000), whereas all other pairwise comparisons between treatments showed significant differences (*P =* 0.006) (Fig. S1A; Table S8). Further investigation using partial dbRDA indicated that antibiotics CTC (*P =* 0.0038) and ENR (*P =* 0.005), as well as *sul1* gene abundance (*P =* 0.0007) and plant biomass (*P =* 0.0001) significantly affected prokaryotic β-diversity (Fig. 1C). Fungal β-diversity was significantly affected by both antibiotic treatment (*P =* 0.001) and plant type (*P =* 0.004). Pairwise comparisons (FDR-adjusted) of the fungal β-diversity showed that no significant differences were observed between c0, c0.1, and c1 (*P >* 0.246), and only c10 was significantly different from the other antibiotic treatments (*P =* 0.006 compared to c0, c0.1, and c1) (Fig. S1B; Table S8). Partial dbRDA for fungal β-diversity revealed significant correlations with plant type (*P =* 0.0009), total plant biomass (*P =* 0.0001), N leaf concentration (*P =* 0.0078), and *sul1* gene abundance (*P =* 0.0006) (Fig. 1F).

**Fig 2:**
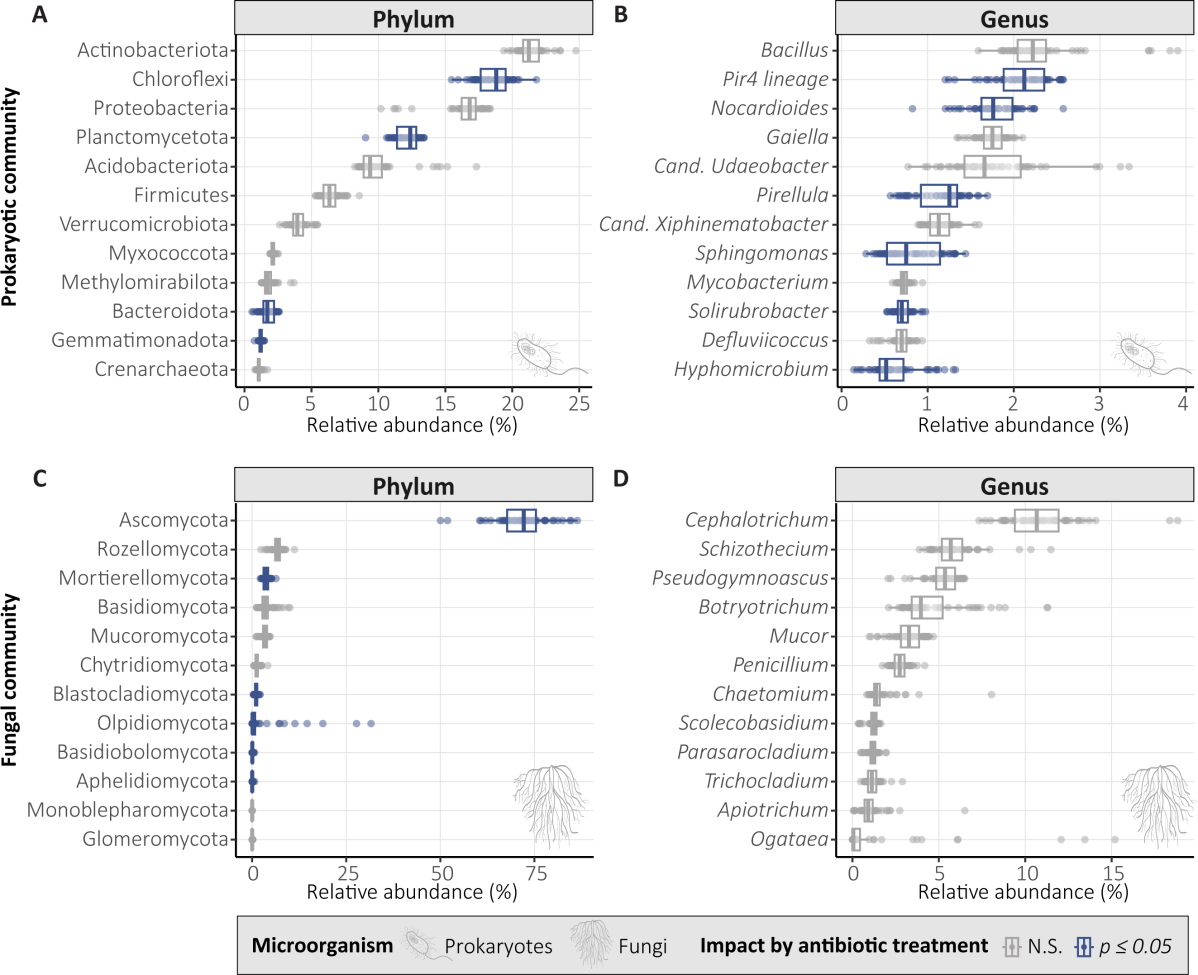
Taxonomic distribution (phyla and genera) of the 12 most abundant prokaryotic phyla (**A**) and genera (**B**) and fungal phyla (**C**) and genera (**D**). Taxonomic groups significantly impacted by antibiotic treatment (PERMANOVA, *P* ≤ 0.05) are colored in blue.

For the prokaryotic community, 13 out of 47 phyla (27.7%) were significantly impacted by antibiotic treatment (*P ≤* 0.05), 4 of which are in the 12 most abundant phyla, namely Chloroflexi (now named Chloroflexota) (*P =* 0.005), Planctomycetota (*P =* 0.005), Bacteroidota (*P =* 0.005), and Gemmatimonadota (*P =* 0.005) (Fig. 2A). At the genus level, 139 out of 671 genera (20.7%) were significantly impacted by antibiotic treatment, affecting 6 of 12 most abundant genera, namely *Pir4* lineage, *Nocardioides*, *Pirellula*, *Sphingomonas*, *Solirubrobacter,* and *Hyphomicrobium* (Fig. 2B). For the fungal community, 6 out of 15 phyla (37.5%) were significantly affected by antibiotic treatment, namely Ascomycota (*P =* 0.04), Aphelidiomycota (*P =* 0.04), Mortierellomycota (*P =* 0.04), Basidiobolomycota (*P =* 0.008), Blastocladiomycota (*P =* 0.008), and Olpidiomycota (*P =* 0.04) (Fig. 2C) and 0 out of 447 genera (Fig. 2D).

### Impact of antibiotics on microbial genera and their characteristics

**Fig 3:**
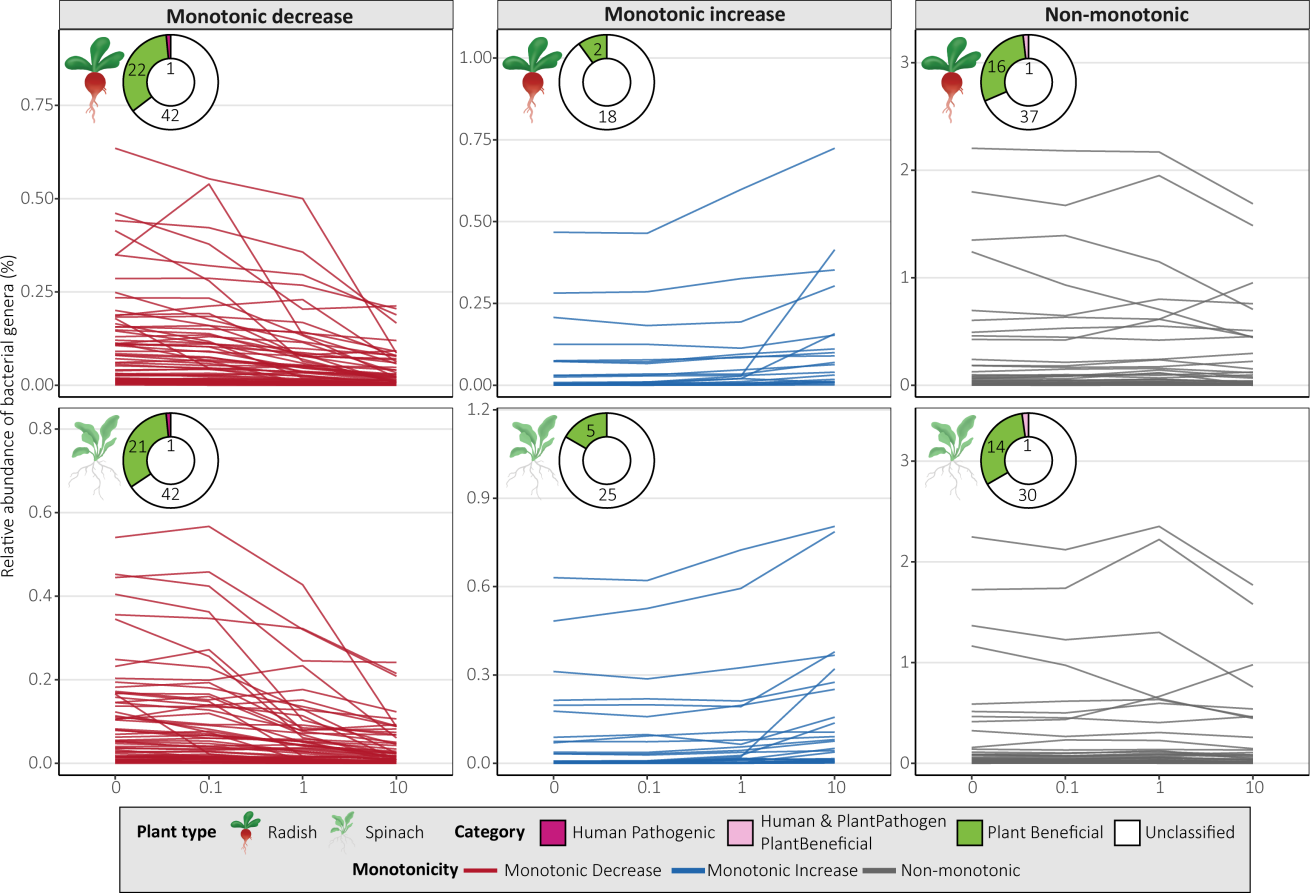
Prokaryotic genera significantly influenced by antibiotic treatment, categorized by monotonic response, with each line representing a genus. Potential microbial categories are classified at the genus level based on the human pathogenic bacteria database (67), the plant-beneficial and pathogenic bacteria database (68); prokaryotes that were not in these databases were labeled as “unclassified.” See also Fig. S2 for the responses of specific genera and their characteristics.

**Fig 4:**
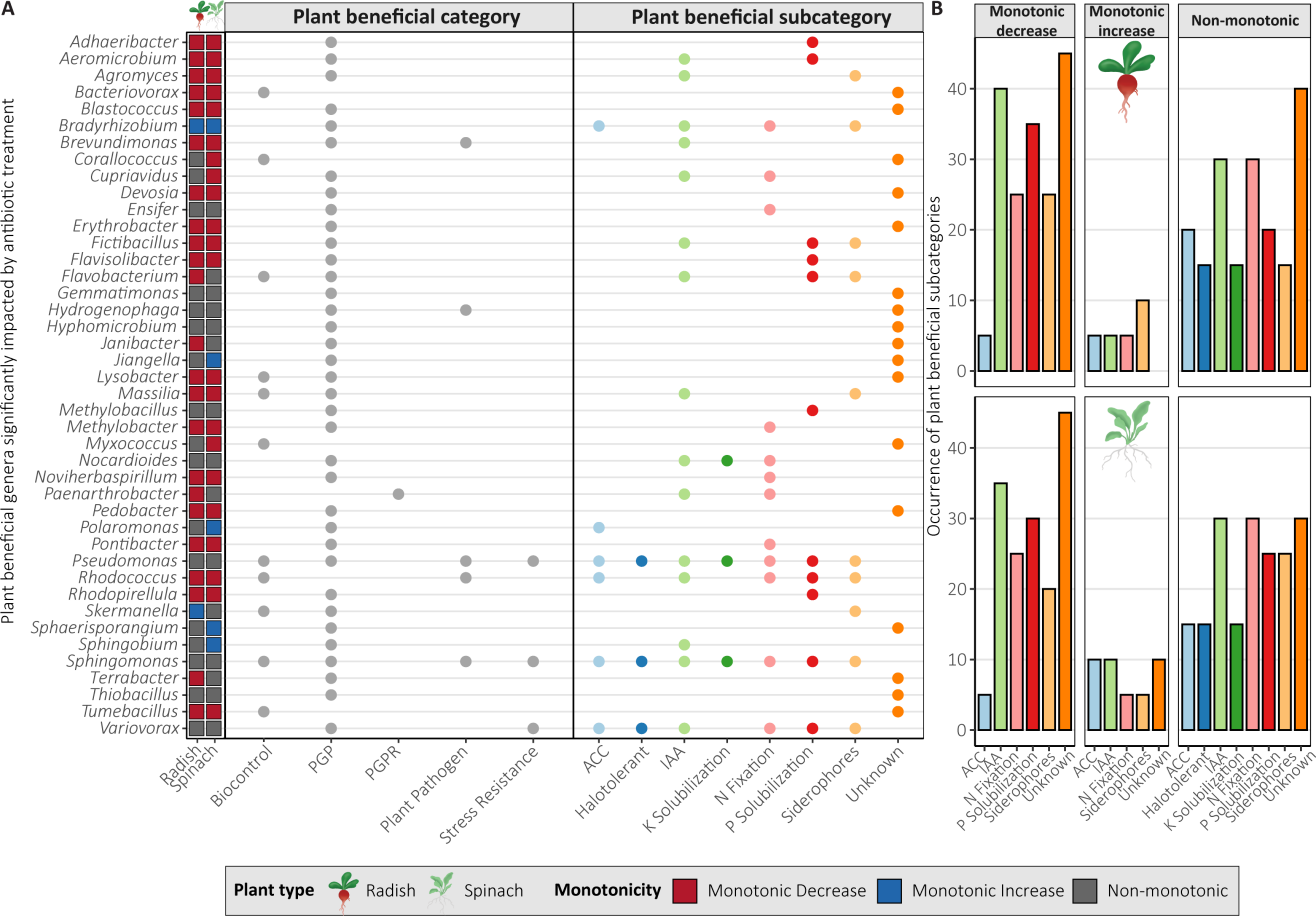
Plant beneficial categories of PGP genera significantly impacted by antibiotic treatment (**A**), and the distribution of the subcategories of these genera by monotonicity group (linear monotonic decrease, linear monotonic increase, and non-monotonic) and plant type (**B**). Plant beneficial categories are based on the plant-beneficial and pathogenic bacteria database (68). Genera that were in the database but did not have a subcategory were categorized as unknown. PGP, plant growth-promoting; PGPR, plant growth-promoting rhizobia; ACC, aminocyclopropane-1-carboxylate deaminase, and IAA, indole-3-acetic acid.

For prokaryotes, 65 (radish, 46.8%) and 64 (spinach, 46.0%) of the 139 genera followed a monotonic decrease, 20 (radish 14.4%) and 30 (spinach, 21.6%) genera followed a monotonic increase, 54 (radish, 38.8%) and 45 (spinach, 32.4%) genera followed a non-monotonic pattern (Fig. 3). Some of the monotonically decreasing genera included known phosphorus-solubilizing genera like *Adhaeribacter* and *Flavisolibacter*, indole-3-acetic acid (IAA)-producing genera like *Aeromicrobium*, *Agromyces,* and *Cupriavidus,* N-fixing genera like *Noviherbaspirillum, Pontibacter,* and *Sphingomonas,* and potentially human pathogenic *Brevundimonas* (Fig. 4A). Monotonically increasing genera included known IAA-producing *Sphingobium*, siderophore-producing *Skermanella*, PGP *Jiangella*, *Polaromonas,* and *Sphaerisporangium*. Many prokaryotic genera followed a non-monotonic pattern, many of which were plant-beneficial, such as N-fixing genera like *Ensifer*, *Pseudomonas,* and *Sphingomonas*, phosphorus-solubilizing genera *Methylobacillus,* and IAA-producing genera *Nocardioides* (Fig. 4A). Some genera followed different patterns depending on plant type but never exhibited both a monotonic decrease and increase (Fig. 4A). In general, the monotonically decreasing genera included mostly IAA-producing, phosphorus-solubilizing, and N-fixing genera, while monotonically increasing had more aminocyclopropane-1-carboxylate deaminase-producing and siderophore-producing genera (Fig. 4B). As no fungal genera were significantly impacted by antibiotic treatment, they were not analyzed for monotonicity.

**Fig 5:**
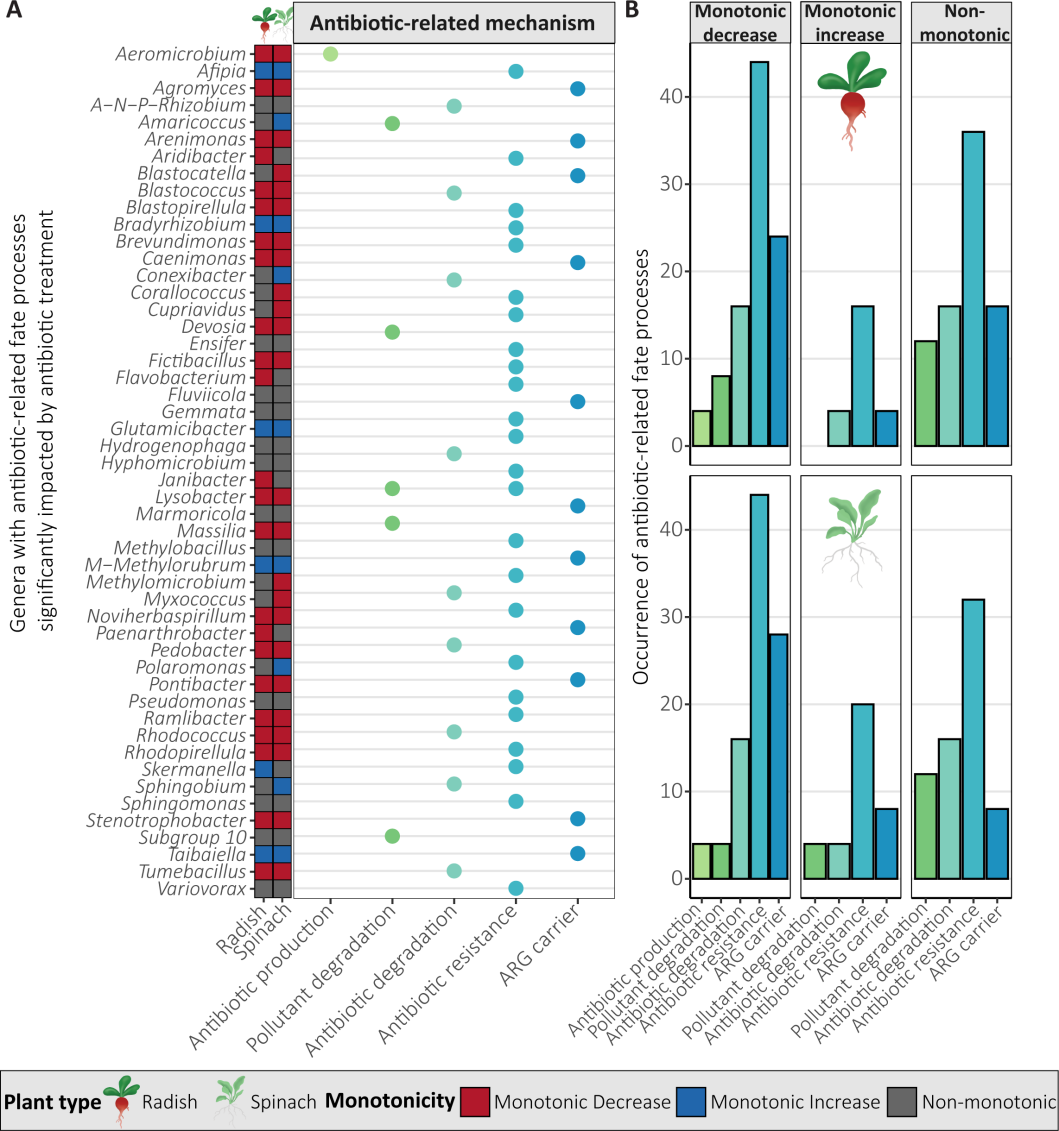
Antibiotic-related fate processes of genera significantly impacted by antibiotic treatment as determined by the CARD database and a literature search (Web of Science), (**A**) and the distribution of the antibiotic-related fate processes of these genera by monotonicity group (linear monotonic decrease, linear monotonic increase, and non-monotonic) and plant type (**B**). A–N–P–*Rhizobium*, *Allorhizobium–Neorhizobium–Pararhizobium–Rhizobium*; M-*Methylorubrum*, *Methylobacter–Methylorubrum*. The results from the CARD database and Web of Science query were published on Figshare (71).

The 139 genera significantly impacted by antibiotic treatment contained one potential antibiotic-producing genus, 5 genera containing potential pollutant degraders, 9 genera containing potential antibiotic degraders, 24 genera containing potential antibiotic-resistant strains, and 11 genera containing ARG carriers (Fig. 5). Many genera, such as *Ensifer*, *Janibacter*, *Pseudomonas,* and *Variovorax,* contain antibiotic-resistant strains (Fig. 5) and did not follow a non-monotonic pattern (Fig. 5A) but increased in c1 compared to c0 (Fig. S2). Other prokaryotic genera following a non-monotonic pattern increased in c0.1 compared to c0, like *Aridibacter*, *Cupriavidus,* and *Myxococcus* (Fig. S2).

### ARGs and MGEs

**Fig 6:**
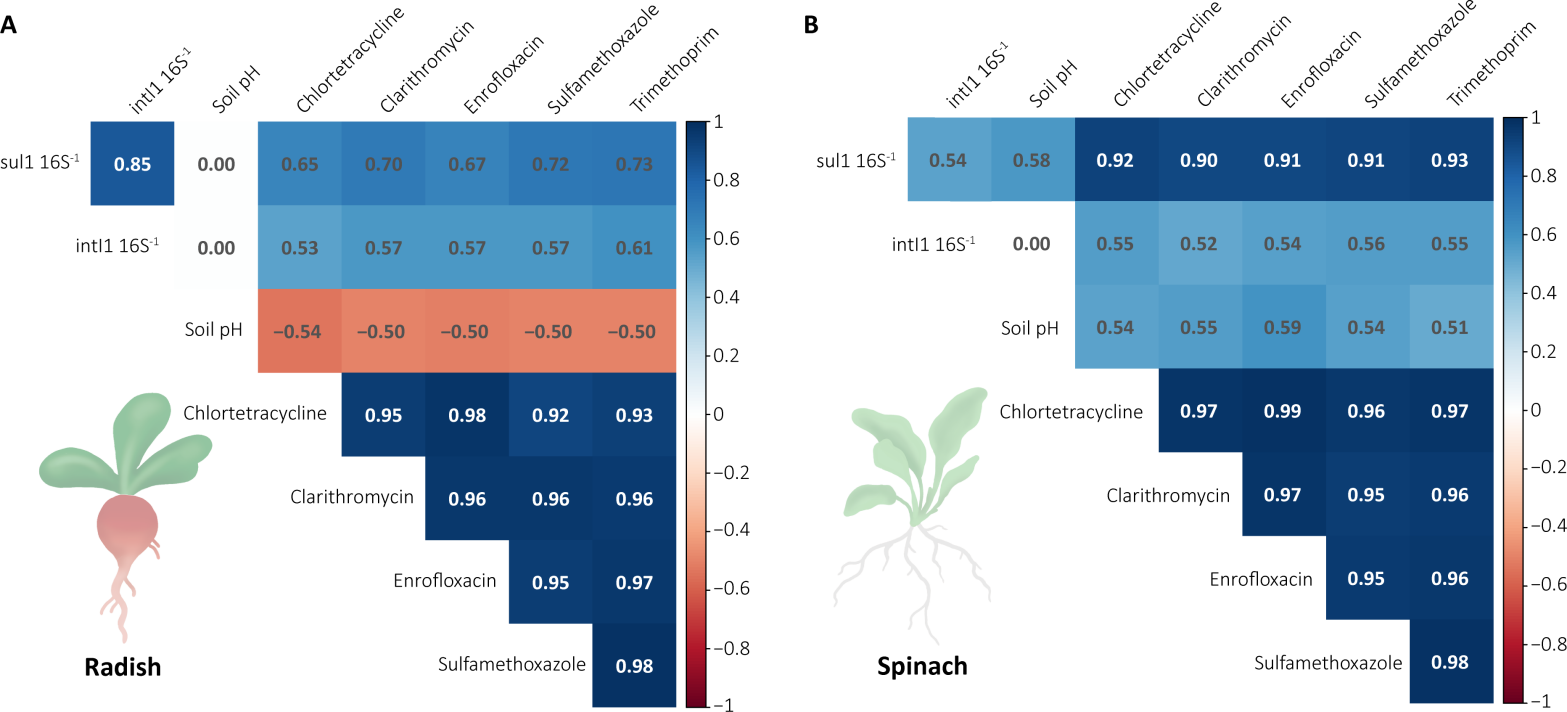
Correlation of *sul1*, *intI1*, soil pH (D42), and measured antibiotic concentrations (D42) across all treatments (c0, c0.1, c1, and c10), separated by plant type for radish (**A**) and spinach (**B**). Correlation coefficient based on Spearman’s *ρ*. Only significant (*P* ≤ 0.05) correlations are shown.

The selected primers can successfully detect ARG *dfrA12*, *tetQ, qnrS1,* and *sul1* and MGE *intI1* and *intI2* according to the *in silico* PCR analysis (Fig. S3). Both *sul1* and *intI1* were present in all samples, while *dfrA12*, *tetQ, qnrS1,* and *intI2* did not amplify with the selected primers and PCR settings. *Sul1* and *intI1* abundance significantly increased in c10 compared to the other antibiotic concentrations, c0, c0.1, and c1. *Sul1* was also higher in c1 for spinach compared to c0 for both plants but not significantly (Fig. S4). A correlation analysis of the genes detected, soil pH, and antibiotic concentration showed that both *sul1* and *intI1* positively correlated with each other in both radish (*Spearman’s ρ =* 0.85) and spinach (*ρ = 0.54*) soils (Fig. 6). *Sul1* was positively correlated with soil pH in the spinach soil samples (*ρ =* 0.58), but not in the radish soil samples. *Sul1* and *intI1* both significantly and positively correlated with the end antibiotic concentrations; for *sul1,* this was particularly strongly correlated for spinach (*ρ =* 0.90–0.93) and to a lesser extent in radish (*ρ =* 0.65–0.72). Soil pH was correlated with the antibiotic treatment for spinach and radish, where c0 showed a significantly higher pH compared to c10 for radish, while the opposite was observed for spinach (Fig. S5). The soil pH of radish and spinach became more similar with increasing antibiotic treatment concentration (Fig. S5), and soil pH was specifically lower for c0 for both radish and spinach, with a soil pH of 7.02 ± 0.06 and 6.71 ± 0.09, respectively (Table S9), compared to the starting soil pH of 7.36 before the sand was added (Table S1). Soil pH also correlated differently with the antibiotic concentrations, ranging from negative for radish (*ρ = −*0.54 to *−*0.50) to positive for spinach (*ρ =* 0.51 to 0.59). For the antibiotics, CTC, CTM, ENR, SMX, and TMP all strongly positively correlated with each other (*ρ >* 0.92).

### Impact of antibiotics on plant biomass and plant nitrogen uptake

**Fig 7:**
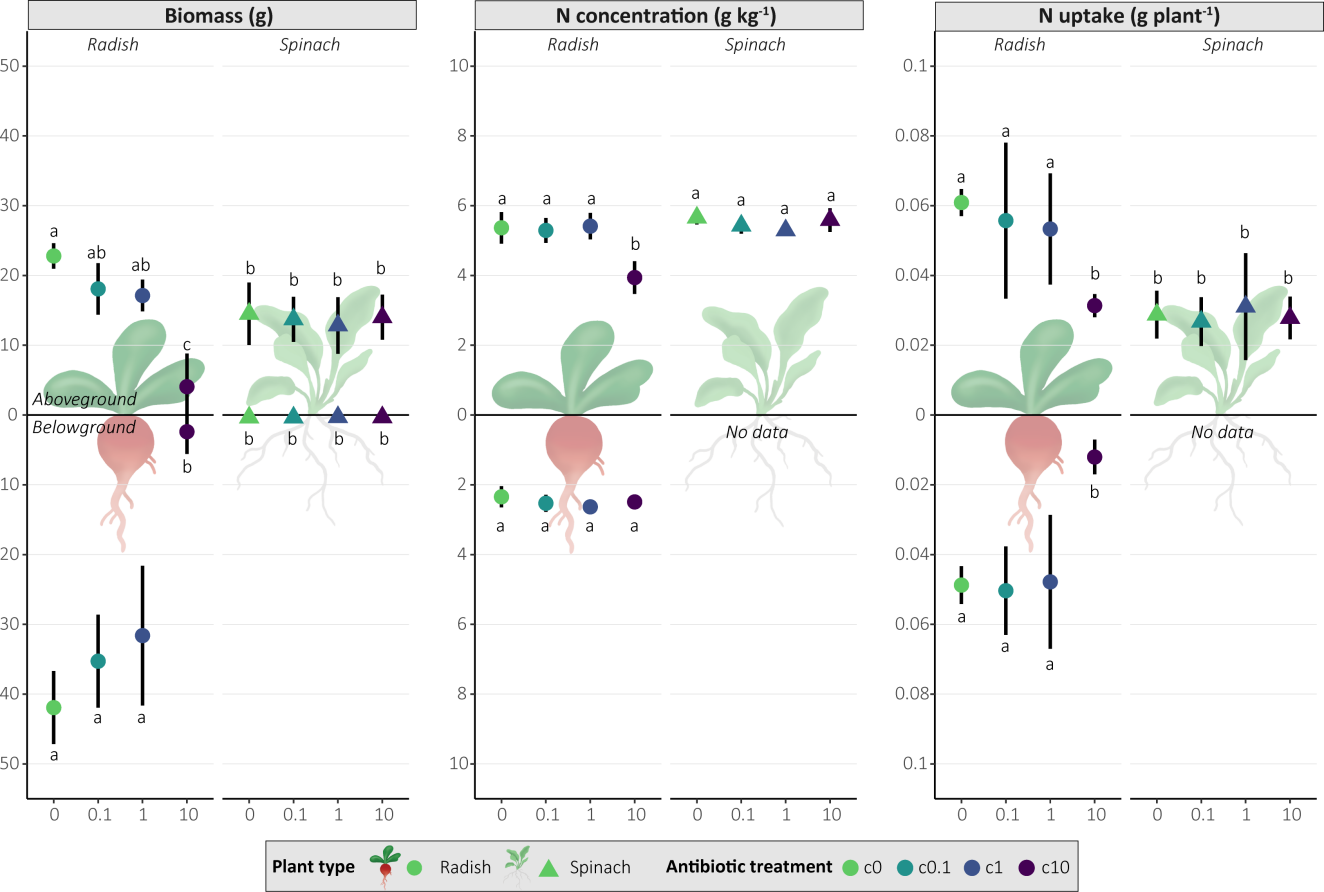
Effect of antibiotic concentration on the absolute values of fresh plant biomass, nitrogen (N) concentration of aboveground and belowground plant tissue, and total N uptake per plant in the leaves (aboveground) and roots (belowground). Differences in letters indicate significant differences between antibiotic treatments and plant types (*P* ≤ 0.05, see also Table S9). Spinach root N was not measured due to insufficient biomass.

The aboveground and belowground biomass of radish decreased significantly with increasing antibiotic concentrations (*P ≤* 0.05), while spinach was not affected (Fig. 7). C10 significantly reduced aboveground N concentration for radish compared to c0, c1, and c10 (*P <* 0.0001), while for spinach, there was no significant effect (*P >* 0.05). Similarly, total N uptake per plant decreased with increasing antibiotic concentrations for radish, but not for spinach (Fig. 7).

## DISCUSSION

### Antibiotic type and concentration drive persistence in soil

The transformation and degradation of antibiotics in soil are driven largely by their molecular structure and physicochemical properties (15). While dissipation of antibiotics varies notably within individual studies, fluoroquinolones, macrolides, and tetracyclines were found to be characterized by long degradation half-lives in soil in a comprehensive review by Cycoń et al. (15). In agreement with this outcome, our results showed that fluoroquinolone ENR and macrolide CLR concentrations remained stable over the course of the experiment (Table 1). This stability is likely facilitated by the measured soil properties, including high silt content and elevated levels of Fe and Al oxides (Table S1). These promote the strong complexation and adsorption of fluoroquinolones and macrolides, consequently reducing their bioavailability for microbial degradation (101, 102). However, the CTC soil concentration at D42 was 25%–42% of that measured initially at D0, indicating favorable conditions for dissipation in our study (Table 1). Degradation half-lives from <1 day to 34 days in soil have been reported in literature for CTC (15). The dissipation of SMX and TMP was most pronounced in our experiment, where at D42 only 12%, 14%, and 16% (SMX) and 3%, 4%, and 45% (TMP) of the initial measured (D0) concentration remained in c0.1, c1, and c10 treatments, respectively (Table 1). Although abiotic degradation processes and other mechanisms of losses (e.g., adsorption to pot surfaces, strong sorption to soil particles leading to the formation of non-extractable residues) cannot be ruled out (see further details in Text S4), it can be assumed that microbial degradation is the primary contributor to the observed dissipation of the antibiotics as indicated by several studies demonstrating that microbes prominently contribute to the degradation of antibiotics in soils (16, 103). However, the highest antibiotic concentration treatment may hinder antibiotic degradation due to the stronger inhibition of soil microbial activity (15, 104, 105). This is reflected in the dissipation of TMP and ENR, where the dissipation for TMP was higher in c0.1 and c1 (≤97%) compared to c10 (≤52%), and the dissipation for ENR c0.1 was 40%, whereas the concentrations remained stable in c1 and c10. Co-application of antibiotics, as done in this experiment, may also lead to inhibition of soil microbial activity and microbial degradation, consequently leading to longer persistence of the antibiotics in soil (106) and continued selection pressure on the soil microbial community.

### Antibiotics exert a stronger influence on prokaryotic diversity than on fungal diversity

Prokaryotic α-diversity significantly reduced with increasing antibiotic concentration (Fig. 1A and B; Table S7), in line with previous research (107). This reduction is likely driven by the high sensitivity of dominant soil taxa. Notably, 4 of the 12 most abundant phyla (Chloroflexota, Planctomycetota, Bacteroidota, and Gemmatimonadota) and half of the 12 most abundant genera (including *Nocardioides* and *Sphingomonas*) were significantly affected. Shifts in these core phyla and genera potentially affect the prokaryotic community’s structural stability. For instance, Bacteroidota and Chloroflexota are typically involved in complex organic matter degradation (108, 109), while *Sphingomonas* and *Nocardioides* are known for their potential use in bioremediation of soils (110, 111). This indicates that antibiotics may impair the soil’s primary metabolic capacities and change the soil’s functioning. Prokaryotic β-diversity was significantly different for c1 and c10 compared to c0 and c0.1 (Table S8). Previous research showed that co-applied antibiotics impact prokaryotic β-diversity (112), but not single antibiotic application (17, 113). Prokaryotic β-diversity was mostly correlated with soil CTC and ENR concentrations, which remained in the soil at high concentrations throughout the experiment (Table 1). As expected, α- and β-diversity were more impacted by antibiotic treatment for the prokaryotic community than the fungal communities (Fig. 1; Table S7), because the five applied antibiotics target bacterial pathways and should have little direct effect on fungi. However, the direct impact of antibiotic exposure on prokaryotic communities and the subsequent changes of abiotic and biotic properties can impact fungal communities. This indirect effect could explain why fungal β-diversity was significantly different in c10 compared to the other antibiotic treatments (Table S8). Thus, we can accept our hypothesis that antibiotic exposure reduces prokaryotic α-diversity, while fungal α-diversity remained unaffected. Similarly, prokaryotic β-diversity was also impacted by antibiotic exposure, while fungal β-diversity was impacted to a lesser extent. These changes have the potential to negatively impact soil and plant health because soil microbial diversity has been shown to be essential in providing ecosystem functions and services for human welfare as a driver of soil fertility and crop productivity (114, 115) and protection against pathogen establishment (116).

### Antibiotic-induced shifts in key bacterial genera threaten microbial-mediated plant support mechanisms

Several bacterial genera were significantly impacted by antibiotic exposure, while no fungal genera were impacted. Only a few bacterial genera were potentially pathogenic, namely *Brevundimonas* (monotonic decrease) and *Pseudomonas* (non-monotonic, but increased at c1), containing both pathogenic and PGP bacteria (117–119) (Fig. 3; Fig. S2). Most responsive PGP bacteria decreased or followed a non-monotonic pattern, potentially affecting N fixation, phosphorus solubilization, production of phytohormone IAA, and plant stress-alleviating enzyme ACC capabilities (Fig. 3 and 4). This suggests that antibiotic exposure may impact plant growth and development through the inhibition of microbial-mediated nutrient cycling, PGP phytohormone production, and stress-alleviating enzymes, which could partially explain reduced plant growth and N uptake for radish in c10 (Fig. 7). Monotonically increasing genera unique to spinach included PGP bacteria and potentially pollutant degraders (Fig. 4 and 5), such as *Amaricoccus* (micropollutant degrader [120]), *Conexibacter* (lincomycin degrader [121]), *Jiangella* (PGP), *Polaromonas* (PGP), *Sphaerisporangium* (PGP), and *Sphingobium* (stress alleviation, biodegradation, and bioremediation [122]) (Fig. 4 and 5). These genera potentially could have contributed to protecting spinach against the effects of antibiotic exposure, thus explaining why it was less affected than radish (Fig. 7).

In our study, most PGP bacteria decreased (Fig. 3 and 4) and only a few PGP bacteria increased in relative abundance and were known antibiotic-resistant or ARG carriers (Fig. 4 and 5), despite the common antibiotic-resistant and stress-adapted nature of PGP bacteria (123–125). This is potentially due to the selective pressure of co-applied antibiotics of different classes. Some responsive genera included tetracycline and sulfonamide degraders, *Hydrophenophaga* (increased at c1 for radish) (126, 127), *Ramlibacter* (unaffected at c0.1 compared to c0) (128–130), and *Skermanella* (increased at c10) (131) (Fig. S2), which likely enhanced CTC and SMX degradation (Table 1). However, co-application of antibiotics may inhibit microbial degradation, especially at high concentrations (see “Antibiotic type and concentration drive persistence in soil,” above). Many significantly impacted genera potentially contain antibiotic-resistant or ARG-carrying species, but few genera increased in relative abundance. Further research is needed to find out which species are antibiotic-resistant and if their ARGs convey resistance to the applied antibiotics. Moreover, it is important to consider that taxonomic shifts do not necessarily translate into altered functional capacities of the soil microbiome (132). Based on our findings, we accept our hypothesis that antibiotic exposure changes taxonomic composition, specifically by reducing PGP bacteria while not affecting fungi. We cannot definitively conclude that antibiotic-resistant microorganisms increased, as only a few genera increased, and thus further analysis is necessary to confirm which species are antibiotic-resistant or ARG-carrying. The reduction of PGP bacterial genera may have significant impacts on nutrient cycling and plant protection mechanisms. Moreover, antibiotic exposure potentially impacted microbial antibiotic degradation and antibiotic resistance, though most genera responded non-monotonically. The genera increasing at low antibiotic concentrations (c0.1 and c1), such as *Pseudomonas,* are potentially the most concerning as these concentrations are more commonly detected in soils amended with organic fertilizers (15, 133). It is therefore especially critical to better understand the impacts of antibiotics at these more common concentrations not only on microbial community structures but also on functional implications for plant productivity and soil health. Therefore, future research utilizing shotgun metagenomics could more precisely determine the impact of these antibiotics on specific PGP capacities, enzyme activities, and nutrient cycling.

### Dose-dependent selection for *sul1* and *intI1* under combined antibiotic pressure

Our study showed a significant increase in *sul1* and *intI1* abundance in the c10 treatment (Fig. S4), likely due to persistent selection pressure. SMX concentration in the c10 treatment remained sufficiently high throughout the experiment to continue selection pressure (Table 1), and both genes were strongly correlated with antibiotic concentrations at D42 (Fig. 6). *Sul1* and *intI1* were not linked to one specific type of antibiotic, as all antibiotics were significantly correlated with these genes (Fig. 6), making it difficult to determine whether one antibiotic exerts more selective pressure than another. We also found a strong correlation between *sul1* and *intI1* (Fig. 6), which is expected because *sul1* lacks a promoter and is always found with *intI1* (78). Additionally, *sul1* and *intI1* were significantly positively correlated with soil pH in spinach but negatively correlated in radish (Fig. 6). This is consistent with previous research, which shows that soil pH (directly or indirectly) impacts ARGs and MGEs (29). In our study, the two plant species created significantly different soil pH (Fig. S5). As antibiotic dissipation did not differ between plants (Table 1), there is likely another driver of soil pH such as plant exudates, which are known to change soil pH (134). A key consideration in interpreting our results is the lack of a positive control, which is important in determining the success of amplification (135). Consequently, we cannot definitively conclude that the lack of amplification for *dfrA12*, *tetQ, qnrS1,* and *intI2* is a true absence or a technical limitation due to primer choice or PCR conditions. Unfortunately, the lack of a positive control is common in studies of ARGs and MGEs due to safety risks and requirements of working with cell cultures harboring ARGs. Nonetheless, we can accept our hypothesis that increasing antibiotic concentrations increases *sul1* and *intI1* abundance. Only c1 (spinach) and c10 (spinach and radish) increased *sul1* and *intI1* abundance (Fig. S4), indicating that lower concentrations may not pose a risk for increasing these genes under combined antibiotic exposure. However, sulfonamides like SMX are regularly used in hospitals as a first- or second-line antibiotic, and *sul1*-mediated resistance to SMX poses a clinical risk when other antibiotics are also rendered ineffective due to antibiotic resistance (136, 137). Therefore, monitoring environmental ARGs is crucial to avoid transfer into clinical settings (27), especially in agricultural settings where consumer crops may be a transfer vehicle for ARGs, and pollution is common.

### Radish exhibits greater susceptibility to antibiotic exposure than spinach

In our study, radish biomass and N uptake were much more affected by antibiotic treatment than spinach (Fig. 7). While some studies have reported no significant effect or even increased radish biomass under single or repeated antibiotic exposure (138–140), our findings align with concentration-dependent responses for radish biomass (141, 142). Previous studies showed that oxytetracycline increased leaf catalase activity, indicative of oxidative stress (139) and could negatively affect radish subcellular structures (chloroplasts and vacuoles) depending on antibiotic type (140). The most significant aboveground and belowground biomass reduction for radish was observed in c10. However, c0.1 and c1, which more closely represent actual environmental concentrations, also had significantly lower aboveground biomass compared to c0. Although the simultaneous presence of five antibiotics at identical concentrations is unlikely due to different degradation rates (see “Antibiotic type and concentration drive persistence in soil,” above), the effects observed at these more realistic exposure levels are important to consider. Antibiotics up to 10 mg kg^−1^ may not affect spinach biomass (143, 144) but can increase oxidative stress (143). In general, plant health response is highly dependent on applied antibiotics (145, 146) and plant type (23, 146), and combined application of antibiotics from different classes may increase toxicity (147). The difference in plant response may be further explained by the plant-dependent reduction of PGP bacteria following antibiotic exposure (Fig. 3 and 4). This can further exacerbate harmful effects on the plants by reducing microbial protection mechanisms (such as oxidative stress protection) and microbial-mediated nutrient cycling. With this, we can accept our hypothesis that increasing antibiotic concentrations decreases plant yield and N uptake for radish but reject our hypothesis for spinach. This highlights the complexity of soil-microbe-plant interactions and emphasizes the need for studies investigating the interplay between microbial activity (i.e., transcriptomics and enzymatic analyses) and plant health under antibiotic exposure.

### Conclusion

This study demonstrates that co-applied antibiotics significantly alter the soil-microbe-plant system, with a larger impact on the prokaryotic community than on the fungal community. While antibiotic exposure reduced prokaryotic α-diversity and changed the composition of plant growth-promoting bacteria, fungal communities remained largely unaffected. The impact of antibiotic exposure increased *sul1* and *intI1*, but not at environmentally relevant concentrations. Moreover, plant yield and nitrogen uptake were highly affected in radish, but not spinach, highlighting the species-specific risks antibiotic exposure poses to plant productivity. In conclusion, these findings suggest that antibiotic exposure may compromise agricultural systems by disrupting nutrient cycling and plant productivity. Future research should focus on disentangling the functional responses of microorganisms to antibiotics on a wider range of plant types to better understand how antibiotic pollution affects environmental health.

## Data Availability

The raw metabarcoding sequences were deposited in the European Nucleotide Archive (ENA) under accession number PRJEB95045. The data used to create Fig. 5 are shared on Figshare under doi https://doi.org/10.6084/m9.figshare.29820431. The scripts used for this project are available on Zenodo under doi https://doi.org/10.5281/zenodo.18048988.

## SUPPLEMENTAL MATERIAL

Supplemental material aem.01581-25-s0001.docx Supplemental text, Tables S1 to S9, and Fig. S1 to S5.

ASM does not own the copyrights to Supplemental Material that may be linked to, or accessed through, an article. The authors have granted ASM a non-exclusive, world-wide license to publish the Supplemental Material files. Please contact the corresponding author directly for reuse.

## References

[1] Gamage A, Gangahagedara R, Gamage J, Jayasinghe N, Kodikara N, Suraweera P, Merah O. 2023. Role of organic farming for achieving sustainability in agriculture. Farming System 1:1–14. doi:10.1016/j.farsys.2023.100005

[2] Trimmer JT, Cusick RD, Guest JS. 2017. Amplifying progress toward multiple development goals through resource recovery from sanitation. Environ Sci Technol 51:10765–10776. doi:10.1021/acs.est.7b02147 28875704

[3] Urra J, Alkorta I, Garbisu C. 2019. Potential benefits and risks for soil health derived from the use of organic amendments in agriculture. Agronomy 9:1–23. doi:10.3390/agronomy9090542

[4] Luo G, Li L, Friman VP, Guo J, Guo S, Shen Q, Ling N. 2018. Organic amendments increase crop yields by improving microbe-mediated soil functioning of agroecosystems: a meta-analysis. Soil Biol Biochem 124:105–115. doi:10.1016/j.soilbio.2018.06.002

[5] Bünemann EK, Reimer M, Smolders E, Smith SR, Bigalke M, Palmqvist A, Brandt KK, Möller K, Harder R, Hermann L, Speiser B, Oudshoorn F, Løes AK, Magid J. 2024. Do contaminants compromise the use of recycled nutrients in organic agriculture? A review and synthesis of current knowledge on contaminant concentrations, fate in the environment and risk assessment. Sci Total Environ 912:1–18. doi:10.1016/j.scitotenv.2023.168901 38042198

[6] van den Broek S, Nybom I, Hartmann M, Doetterl S, Garland G. 2024. Opportunities and challenges of using human excreta-derived fertilizers in agriculture: a review of suitability, environmental impact and societal acceptance. Sci Total Environ 957:1–21. doi:10.1016/j.scitotenv.2024.177306 39515389

[7] Barathe P, Kaur K, Reddy S, Shriram V, Kumar V. 2024. Antibiotic pollution and associated antimicrobial resistance in the environment. J Hazard Mater Lett 5:1–11. doi:10.1016/j.hazl.2024.100105

[8] Frey L, Tanunchai B, Glaser B. 2022. Antibiotics residues in pig slurry and manure and its environmental contamination potential. A meta-analysis. Agron Sustain Dev 42:1–10. doi:10.1007/s13593-022-00762-y

[9] Wu J, Wang J, Li Z, Guo S, Li K, Xu P, Ok YS, Jones DL, Zou J. 2023. Antibiotics and antibiotic resistance genes in agricultural soils: a systematic analysis. Crit Rev Environ Sci Technol 53:847–864. doi:10.1080/10643389.2022.2094693

[10] Yang Q, Gao Y, Ke J, Show PL, Ge Y, Liu Y, Guo R, Chen J. 2021. Antibiotics: an overview on the environmental occurrence, toxicity, degradation, and removal methods. Bioengineered 12:7376–7416. doi:10.1080/21655979.2021.1974657 34612807 PMC8806427

[11] Chen C, Li J, Chen P, Ding R, Zhang P, Li X. 2014. Occurrence of antibiotics and antibiotic resistances in soils from wastewater irrigation areas in Beijing and Tianjin, China. Environ Pollut 193:94–101. doi:10.1016/j.envpol.2014.06.005 25016103

[12] Tian L, Sun H, Dong X, Wang J, Huang Y, Sun S. 2022. Effects of swine wastewater irrigation on soil properties and accumulation of heavy metals and antibiotics. J Soils Sediments 22:889–904. doi:10.1007/s11368-021-03106-7

[13] Kümmerer K. 2009. Antibiotics in the aquatic environment – A review – Part I. Chemosphere 75:417–434. doi:10.1016/j.chemosphere.2008.11.086 19185900

[14] Halawa EM, Fadel M, Al-Rabia MW, Behairy A, Nouh NA, Abdo M, Olga R, Fericean L, Atwa AM, El-Nablaway M, Abdeen A. 2023. Antibiotic action and resistance: updated review of mechanisms, spread, influencing factors, and alternative approaches for combating resistance. Front Pharmacol 14:1–17. doi:10.3389/fphar.2023.1305294 PMC10820715 38283841

[15] Cycoń M, Mrozik A, Piotrowska-Seget Z. 2019. Antibiotics in the soil environment—degradation and their impact on microbial activity and diversity. Front Microbiol 10:1–45. doi:10.3389/fmicb.2019.00338 30906284 PMC6418018

[16] Pan M, Chu LM. 2016. Phytotoxicity of veterinary antibiotics to seed germination and root elongation of crops. Ecotoxicol Environ Saf 126:228–237. doi:10.1016/j.ecoenv.2015.12.027 26773832

[17] Cleary DW, Bishop AH, Zhang L, Topp E, Wellington EMH, Gaze WH. 2016. Long-term antibiotic exposure in soil is associated with changes in microbial community structure and prevalence of class 1 integrons. FEMS Microbiol Ecol 92:1–7. doi:10.1093/femsec/fiw159 27495240

[18] Chen J, Zhu B, Zhang Y. 2023. A meta-analysis on the responses of soil microbial biomass and community structure to antibiotics. Appl Soil Ecol 184:1–11. doi:10.1016/j.apsoil.2022.104786

[19] Arseneault T, Filion M. 2017. Biocontrol through antibiosis: exploring the role played by subinhibitory concentrations of antibiotics in soil and their impact on plant pathogens. Can J Plant Pathol 39:267–274. doi:10.1080/07060661.2017.1354335

[20] Ren J, Lu H, Lu S, Huang Z. 2024. Impacts of sulfamethoxazole stress on vegetable growth and rhizosphere bacteria and the corresponding mitigation mechanism. Front Bioeng Biotechnol 12:1–11. doi:10.3389/fbioe.2024.1303670 PMC10882545 38390364

[21] Ding GC, Radl V, Schloter-Hai B, Jechalke S, Heuer H, Smalla K, Schloter M. 2014. Dynamics of soil bacterial communities in response to repeated application of manure containing sulfadiazine. PLoS One 9:1–10. doi:10.1371/journal.pone.0092958 PMC3966856 24671113

[22] Krupka M, Piotrowicz-Cieślak AI, Michalczyk DJ. 2022. Effects of antibiotics on the photosynthetic apparatus of plants. J Plant Interact 17:96–104. doi:10.1080/17429145.2021.2014579

[23] Minden V, Deloy A, Volkert AM, Leonhardt SD, Pufal G. 2017. Antibiotics impact plant traits, even at small concentrations. AoB Plants 9:1–19. doi:10.1093/aobpla/plx010 PMC5393049 28439396

[24] Li L, Li T, Liu Y, Li L, Huang X, Xie J. 2023. Effects of antibiotics stress on root development, seedling growth, antioxidant status and abscisic acid level in wheat (Triticum aestivum L.). Ecotoxicol Environ Saf 252:1–10. doi:10.1016/j.ecoenv.2023.114621 36774794

[25] Carballo M, Rodríguez A, de la Torre A. 2022. Phytotoxic effects of antibiotics on terrestrial crop plants and wild plants: a systematic review. Arch Environ Contam Toxicol 82:48–61. doi:10.1007/s00244-021-00893-5 34671816 PMC8732949

[26] Fiaz M, Ahmed I, Hassan SMU, Niazi AK, Khokhar MF, Farooq MA, Arshad M, Zeshan. 2023. Antibiotics induced changes in nitrogen metabolism and antioxidative enzymes in mung bean (Vigna radiata). Sci Total Environ 873:1–10. doi:10.1016/j.scitotenv.2023.162449 36841411

[27] Larsson DGJ, Flach CF. 2022. Antibiotic resistance in the environment. Nat Rev Microbiol 20:257–269. doi:10.1038/s41579-021-00649-x 34737424 PMC8567979

[28] Gattinger D, Schlenz V, Weil T, Sattler B. 2024. From remote to urbanized: dispersal of antibiotic-resistant bacteria under the aspect of anthropogenic influence. Sci Total Environ 924:1–11. doi:10.1016/j.scitotenv.2024.171532 38458439

[29] Delgado-Baquerizo M, Hu H-W, Maestre FT, Guerra CA, Eisenhauer N, Eldridge DJ, Zhu Y-G, Chen Q-L, Trivedi P, Du S, et al.. 2022. The global distribution and environmental drivers of the soil antibiotic resistome. Microbiome 10:1–15. doi:10.1186/s40168-022-01405-w 36503688 PMC9743735

[30] Laureti L, Matic I, Gutierrez A. 2013. Bacterial responses and genome instability induced by subinhibitory concentrations of antibiotics. Antibiotics (Basel) 2:100–114. doi:10.3390/antibiotics2010100 27029295 PMC4790301

[31] Buffie CG, Pamer EG. 2013. Microbiota-mediated colonization resistance against intestinal pathogens. Nat Rev Immunol 13:790–801. doi:10.1038/nri3535 24096337 PMC4194195

[32] Lau CHF, Tien YC, Stedtfeld RD, Topp E. 2020. Impacts of multi-year field exposure of agricultural soil to macrolide antibiotics on the abundance of antibiotic resistance genes and selected mobile genetic elements. Sci Total Environ 727:1–7. doi:10.1016/j.scitotenv.2020.138520 32330714

[33] Larsson DGJ, Andremont A, Bengtsson-Palme J, Brandt KK, de Roda Husman AM, Fagerstedt P, Fick J, Flach C-F, Gaze WH, Kuroda M, et al.. 2018. Critical knowledge gaps and research needs related to the environmental dimensions of antibiotic resistance. Environ Int 117:132–138. doi:10.1016/j.envint.2018.04.041 29747082

[34] Peters AC, Larsson DGJ, Laxminarayan R, Munthe C. 2024. Barriers and pathways to environmental surveillance of antibiotic resistance in middle- and low-income settings: a qualitative exploratory key expert study. Glob Health Action 17:1–16. doi:10.1080/16549716.2024.2343318 PMC11141306 38813982

[35] Waglechner N, Wright GD. 2017. Antibiotic resistance: it’s bad, but why isn’t it worse? BMC Biol 15:1–8. doi:10.1186/s12915-017-0423-1 28915805 PMC5603022

[36] Zhou SYD, Wei MY, Giles M, Neilson R, Zheng F, Zhang Q, Zhu YG, Yang XR. 2020. Prevalence of antibiotic resistome in ready-to-eat salad. Front Public Health 8:1–9. doi:10.3389/fpubh.2020.00092 32269985 PMC7109403

[37] Ransirini AM, Elżbieta MS, Joanna G, Bartosz K, Wojciech T, Agnieszka B, Magdalena U. 2024. Fertilizing drug resistance: dissemination of antibiotic resistance genes in soil and plant bacteria under bovine and swine slurry fertilization. Sci Total Environ 946:1–15. doi:10.1016/j.scitotenv.2024.174476 38969119

[38] Richter L, du Plessis EM, Duvenage S, Korsten L. 2020. Occurrence, phenotypic and molecular characterization of extended-spectrum- and AmpC- β-lactamase producing Enterobacteriaceae isolated from selected commercial spinach supply chains in South Africa. Front Microbiol 11:1–10. doi:10.3389/fmicb.2020.00638 32351477 PMC7176360

[39] Sarmah AK, Meyer MT, Boxall ABA. 2006. A global perspective on the use, sales, exposure pathways, occurrence, fate and effects of veterinary antibiotics (VAs) in the environment. Chemosphere 65:725–759. doi:10.1016/j.chemosphere.2006.03.026 16677683

[40] Stephens CR, Murai K, Brunings KJ, Woodward RB. 1956. Acidity constants of the tetracycline antibiotics. J Am Chem Soc 78:4155–4158. doi:10.1021/ja01597a081

[41] Nowara A, Burhenne J, Spiteller M. 1997. Binding of fluoroquinolone carboxylic acid derivatives to clay minerals. J Agric Food Chem 45:1459–1463. doi:10.1021/jf960215l

[42] Boxall ABA, Johnson P, Smith EJ, Sinclair CJ, Stutt E, Levy LS. 2006. Uptake of veterinary medicines from soils into plants. J Agric Food Chem 54:2288–2297. doi:10.1021/jf053041t 16536609

[43] McFarland JW, Berger CM, Froshauer SA, Hayashi SF, Hecker SJ, Jaynes BH, Jefson MR, Kamicker BJ, Lipinski CA, Lundy KM, Reese CP, Vu CB. 1997. Quantitative structure-activity relationships among macrolide antibacterial agents: in vitro and in vivo potency against Pasteurella multocida. J Med Chem 40:1340–1346. doi:10.1021/jm960436i 9135031

[44] Lin K, Gan J. 2011. Sorption and degradation of wastewater-associated non-steroidal anti-inflammatory drugs and antibiotics in soils. Chemosphere 83:240–246. doi:10.1016/j.chemosphere.2010.12.083 21247615

[45] Avisar D, Primor O, Gozlan I, Mamane H. 2010. Sorption of sulfonamides and tetracyclines to montmorillonite clay. Water Air Soil Pollut 209:439–450. doi:10.1007/s11270-009-0212-8

[46] Stoob K, Singer HP, Mueller SR, Schwarzenbach RP, Stamm CH. 2007. Dissipation and transport of veterinary sulfonamide antibiotics after manure application to grassland in a small catchment. Environ Sci Technol 41:7349–7355. doi:10.1021/es070840e 18044510

[47] Dean A, Morris M, Stufken J, Bingham D. 2015. Handbook of design and analysis of experiments. Chapman and Hall, New York.

[48] Shi X, Zhang S, Zhang Y, Geng Y, Wang L, Peng Y, He Z. 2022. Novel and simple analytical method for simultaneous determination of sulfonamide, quinolone, tetracycline, macrolide, and chloramphenicol antibiotics in soil. Anal Bioanal Chem 414:6497–6506. doi:10.1007/s00216-022-04206-0 35829769

[49] Frey B, Rime T, Phillips M, Stierli B, Hajdas I, Widmer F, Hartmann M. 2016. Microbial diversity in European alpine permafrost and active layers. FEMS Microbiol Ecol 92:1–17. doi:10.1093/femsec/fiw018 26832204

[50] Tedersoo L, Lindahl B. 2016. Fungal identification biases in microbiome projects. Environ Microbiol Rep 8:774–779. doi:10.1111/1758-2229.12438 27348848

[51] Longepierre M, Widmer F, Keller T, Weisskopf P, Colombi T, Six J, Hartmann M. 2021. Limited resilience of the soil microbiome to mechanical compaction within four growing seasons of agricultural management. ISME Commun 1:1–13. doi:10.1038/s43705-021-00046-8 36740718 PMC9723577

[52] Andrews S. 2010. FastQC: a quality control tool for high throughput sequence data. Babraham Bioinformatics, Babraham Institute, Cambridge, UK. https://www.bioinformatics.babraham.ac.uk/projects/fastqc/.

[53] Rognes T, Flouri T, Nichols B, Quince C, Mahé F. 2016. VSEARCH: a versatile open source tool for metagenomics. PeerJ 4:1–22. doi:10.7717/peerj.2584 PMC5075697 27781170

[54] Langmead B, Salzberg SL. 2012. Fast gapped-read alignment with Bowtie 2. Nat Methods 9:357–359. doi:10.1038/nmeth.1923 22388286 PMC3322381

[55] Martin M. 2011. Cutadapt removes adapter sequences from high-throughput sequencing reads. EMBnet J 17:10–12. doi:10.14806/ej.17.1.200

[56] Chen S, Zhou Y, Chen Y, Gu J. 2018. Fastp: an ultra-fast all-in-one FASTQ preprocessor. Bioinformatics 34:i884–i890. doi:10.1093/bioinformatics/bty560 30423086 PMC6129281

[57] Edgar RC. 2016. UNOISE2: improved error-correction for Illumina 16S and ITS amplicon sequencing. bioRxiv. doi:10.1101/081257

[58] Edgar RC. 2016. UCHIME2: improved chimera prediction for amplicon sequencing. bioRxiv. doi:10.1101/074252

[59] Bengtsson-Palme J, Hartmann M, Eriksson KM, Pal C, Thorell K, Larsson DGJ, Nilsson RH. 2015. metaxa2: improved identification and taxonomic classification of small and large subunit rRNA in metagenomic data. Mol Ecol Resour 15:1403–1414. doi:10.1111/1755-0998.12399 25732605

[60] Bengtsson‐Palme J, Ryberg M, Hartmann M, Branco S, Wang Z, Godhe A, De Wit P, Sánchez‐García M, Ebersberger I, de Sousa F, Amend A, Jumpponen A, Unterseher M, Kristiansson E, Abarenkov K, Bertrand YJK, Sanli K, Eriksson KM, Vik U, Veldre V, Nilsson RH. 2013. Improved software detection and extraction of ITS1 and ITS2 from ribosomal ITS sequences of fungi and other eukaryotes for analysis of environmental sequencing data. Methods Ecol Evol 4:914–919. doi:10.1111/2041-210X.12073

[61] Pruesse E, Quast C, Knittel K, Fuchs BM, Ludwig W, Peplies J, Glöckner FO. 2007. SILVA: a comprehensive online resource for quality checked and aligned ribosomal RNA sequence data compatible with ARB. Nucleic Acids Res 35:7188–7196. doi:10.1093/nar/gkm864 17947321 PMC2175337

[62] Abarenkov K, Nilsson RH, Larsson KH, Taylor AFS, May TW, Frøslev TG, Pawlowska J, Lindahl B, Põldmaa K, Truong C, et al.. 2024. The UNITE database for molecular identification and taxonomic communication of fungi and other eukaryotes: sequences, taxa and classifications reconsidered. Nucleic Acids Res 52:D791–D797. doi:10.1093/nar/gkad1039 37953409 PMC10767974

[63] Edgar RC. 2016. SINTAX: a simple non-Bayesian taxonomy classifier for 16S and ITS sequences. bioRxiv. doi:10.1101/074161

[64] Wickham H, Henry L, Müller K. 2017. A grammar of data manipulation. R Foundation for Statistical Computing, Vienna, Austria. https://CRAN.R-project.org/package=dplyr.

[65] RStudio Team. 2020. RStudio: integrated development for R. RStudio, PBC, Boston, MA.

[66] R Core Team. 2021. A language and environment for statistical computing. R Foundation for Statistical Computing, Vienna, Austria.

[67] Bartlett A, Padfield D, Lear L, Bendall R, Vos M. 2022. A comprehensive list of bacterial pathogens infecting humans. Microbiology (Reading, Engl) 168:1–8. doi:10.1099/mic.0.001269 36748702

[68] Li P, Tedersoo L, Crowther TW, Dumbrell AJ, Dini-Andreote F, Bahram M, Kuang L, Li T, Wu M, Jiang Y, Luan L, Saleem M, de Vries FT, Li Z, Wang B, Jiang J. 2023. Fossil-fuel-dependent scenarios could lead to a significant decline of global plant-beneficial bacteria abundance in soils by 2100. Nat Food 4:996–1006. doi:10.1038/s43016-023-00869-9 37904026

[69] Nguyen NH, Song Z, Bates ST, Branco S, Tedersoo L, Menke J, Schilling JS, Kennedy PG. 2016. FUNGuild: an open annotation tool for parsing fungal community datasets by ecological guild. Fungal Ecol 20:241–248. doi:10.1016/j.funeco.2015.06.006

[70] Alcock BP, Raphenya AR, Lau TTY, Tsang KK, Bouchard M, Edalatmand A, Huynh W, Nguyen A-LV, Cheng AA, Liu S, et al.. 2020. CARD 2020: antibiotic resistome surveillance with the comprehensive antibiotic resistance database. Nucleic Acids Res 48:D517–D525. doi:10.1093/nar/gkz935 31665441 PMC7145624

[71] Sun Y, F, Bucheli TD, Doetterl S, Martin Hartmann M, Garland G. 2025. Table_Significant_Genera_AntibioticProcesses.xlsx. Figshare. 10.6084/m9.figshare.29820431.

[72] Bischel HN, Özel Duygan BD, Strande L, McArdell CS, Udert KM, Kohn T. 2015. Pathogens and pharmaceuticals in source-separated urine in eThekwini, South Africa. Water Res 85:57–65. doi:10.1016/j.watres.2015.08.022 26302215

[73] Burch TR, Sadowsky MJ, LaPara TM. 2017. Effect of different treatment technologies on the fate of antibiotic resistance genes and class 1 integrons when residual municipal wastewater solids are applied to soil. Environ Sci Technol 51:14225–14232. doi:10.1021/acs.est.7b04760 29148730

[74] Xie WY, McGrath SP, Su JQ, Hirsch PR, Clark IM, Shen Q, Zhu YG, Zhao FJ. 2016. Long-term impact of field applications of sewage sludge on soil antibiotic resistome. Environ Sci Technol 50:12602–12611. doi:10.1021/acs.est.6b02138 27934260

[75] Liao H, Lu X, Rensing C, Friman VP, Geisen S, Chen Z, Yu Z, Wei Z, Zhou S, Zhu Y. 2018. Hyperthermophilic composting accelerates the removal of antibiotic resistance genes and mobile genetic elements in sewage sludge. Environ Sci Technol 52:266–276. doi:10.1021/acs.est.7b04483 29199822

[76] Zalewska M, Błażejewska A, Czapko A, Popowska M. 2021. Antibiotics and antibiotic resistance genes in animal manure – consequences of its application in agriculture. Front Microbiol 12:1–21. doi:10.3389/fmicb.2021.610656 PMC8039466 33854486

[77] Liu W, Li B, Chu H, Zhang Z, Luo L, Ma W, Yang S, Guo Q. 2017. Rapid detection of mutations in erm(41) and rrl associated with clarithromycin resistance in Mycobacterium abscessus complex by denaturing gradient gel electrophoresis. J Microbiol Methods 143:87–93. doi:10.1016/j.mimet.2017.10.010 29079298

[78] de Los Santos E, Laviña M, Poey ME. 2021. Strict relationship between class 1 integrons and resistance to sulfamethoxazole in Escherichia coli. Microb Pathog 161:1–6. doi:10.1016/j.micpath.2021.105206 34619311

[79] Hansson K, Sundström L, Pelletier A, Roy PH. 2002. IntI2 integron integrase in Tn7. J Bacteriol 184:1712–1721. doi:10.1128/JB.184.6.1712-1721.2002 11872723 PMC134885

[80] Kerrn MB, Klemmensen T, Frimodt-Møller N, Espersen F. 2002. Susceptibility of Danish Escherichia coli strains isolated from urinary tract infections and bacteraemia, and distribution of sul genes conferring sulphonamide resistance. J Antimicrob Chemother 50:513–516. doi:10.1093/jac/dkf164 12356795

[81] Marti E, Balcázar JL. 2013. Real-time PCR assays for quantification of qnr genes in environmental water samples and chicken feces. Appl Environ Microbiol 79:1743–1745. doi:10.1128/AEM.03409-12 23275512 PMC3591933

[82] Rosewarne CP, Pettigrove V, Stokes HW, Parsons YM. 2010. Class 1 integrons in benthic bacterial communities: abundance, association with Tn402-like transposition modules and evidence for coselection with heavy-metal resistance. FEMS Microbiol Ecol 72:35–46. doi:10.1111/j.1574-6941.2009.00823.x 20132306

[83] Luo Y, Mao D, Rysz M, Zhou Q, Zhang H, Xu L, J J Alvarez P. 2010. Trends in antibiotic resistance genes occurrence in the Haihe River, China. Environ Sci Technol 44:7220–7225. doi:10.1021/es100233w 20509603

[84] Hu HW, Wang JT, Li J, Li JJ, Ma YB, Chen D, He JZ. 2016. Field‐based evidence for copper contamination induced changes of antibiotic resistance in agricultural soils. Environ Microbiol 18:3896–3909. doi:10.1111/1462-2920.13370 27207327

[85] National Center for Biotechnology Information. 2004. Gene. Bethesda (MD) National Library of Medicine (US), National Center for Biotechnology Information

[86] Charif D, Lobry JR. 2004. seqinr: biological sequences retrieval and analysis. CRAN Contrib Packag. Available from: 10.32614/CRAN.PACKAGE.SEQINR

[87] Wickham H, Chang W. 2016. Create elegant data visualisations using the grammar of graphics

[88] Pedersen TL. 2025. patchwork: the composer of plots. R package version 1.3.0.9000. https://github.com/thomasp85/patchwork.

[89] Neuwirth E. 2011. Package “RColorBrewer”. Phys Rev D - Part Fields, Gravit Cosmol 84

[90] Adobe Inc. 2019. Adobe Illustrator. 29.4. San Jose, CA Adobe Inc.

[91] John F, Weisberg S. 2019. An R Companion to Applied Regression. Sage. https://www.john-fox.ca/Companion/.

[92] Hothorn T, Zeileis A, Farebrother RW, Cummins C. 2022. Testing linear regression models. R package lmtest version 0.9-40. CRAN Contrib Packag. 10.32614/CRAN.PACKAGE.LMTEST.

[93] Kassambara A. 2025. Pipe-friendly framework for basic statistical tests. R package rstatix version 0.7.3. CRAN Contrib Packag. 10.32614/CRAN.PACKAGE.RSTATIX.

[94] Graves S, Piepho H-P, Selzer L. 2024. multcompView: visualizations of paired comparisons. https://CRAN.R‑project.org/package=multcompView.

[95] Schloss PD. 2024. Rarefaction is currently the best approach to control for uneven sequencing effort in amplicon sequence analyses. mSphere 9:1–20. doi:10.1128/msphere.00354-23 PMC10900887 38251877

[96] Schloss PD. 2024. Waste not, want not: revisiting the analysis that called into question the practice of rarefaction. mSphere 9:1–20. doi:10.1128/msphere.00355-23 PMC10826360 38054712

[97] Oksanen AJ, Blanchet FG, Friendly M, Kindt R, Legendre P, Mcglinn D, Minchin PR, Hara RBO, Simpson GL, Solymos P, Stevens MHH, Szoecs E. 2012. vegan: community ecology package

[98] Martinez Arbizu P. 2020. pairwiseAdonis: pairwise multilevel comparison using adonis. R package version 0.4. https://CRAN.R-project.org/package=pairwiseAdonis.

[99] Harrell JF. 2025. Hmisc: harrell miscellaneous. R package version 5.2-4. https://CRAN.R-project.org/package=Hmisc.

[100] Wei T, Simko V. 2024. R package “corrplot”: visualization of a correlation matrix. R package version 0.95. https://CRAN.R-project.org/package=corrplot.

[101] Zhang Y, Cheng D, Xie J, Zhang Y, Wan Y, Zhang Y, Shi X. 2022. Impacts of farmland application of antibiotic-contaminated manures on the occurrence of antibiotic residues and antibiotic resistance genes in soil: a meta-analysis study. Chemosphere 300:1–13. doi:10.1016/j.chemosphere.2022.134529 35395269

[102] Jechalke S, Heuer H, Siemens J, Amelung W, Smalla K. 2014. Fate and effects of veterinary antibiotics in soil. Trends Microbiol 22:536–545. doi:10.1016/j.tim.2014.05.005 24950802

[103] Yang JF, Ying GG, Zhou LJ, Liu S, Zhao JL. 2009. Dissipation of oxytetracycline in soils under different redox conditions. Environ Pollut 157:2704–2709. doi:10.1016/j.envpol.2009.04.031 19467565

[104] Demoling LA, Bååth E, Greve G, Wouterse M, Schmitt H. 2009. Effects of sulfamethoxazole on soil microbial communities after adding substrate. Soil Biol Biochem 41:840–848. doi:10.1016/j.soilbio.2009.02.001

[105] Fang H, Han L, Cui Y, Xue Y, Cai L, Yu Y. 2016. Changes in soil microbial community structure and function associated with degradation and resistance of carbendazim and chlortetracycline during repeated treatments. Sci Total Environ 572:1203–1212. doi:10.1016/j.scitotenv.2016.08.038 27524727

[106] Kodešová R, Chroňáková A, Grabicová K, Kočárek M, Schmidtová Z, Frková Z, Vojs Staňová A, Nikodem A, Klement A, Fér M, Grabic R. 2020. How microbial community composition, sorption and simultaneous application of six pharmaceuticals affect their dissipation in soils. Sci Total Environ 746:1–14. doi:10.1016/j.scitotenv.2020.141134 32768780

[107] Gao Q, Gao S, Bates C, Zeng Y, Lei J, Su H, Dong Q, Qin Z, Zhao J, Zhang Q, Ning D, Huang Y, Zhou J, Yang Y. 2021. The microbial network property as a bio-indicator of antibiotic transmission in the environment. Sci Total Environ 758:1–10. doi:10.1016/j.scitotenv.2020.143712 33277004

[108] Thomas F, Hehemann JH, Rebuffet E, Czjzek M, Michel G. 2011. Environmental and gut bacteroidetes: the food connection. Front Microbiol 2:1–16. doi:10.3389/fmicb.2011.00093 21747801 PMC3129010

[109] Freches A, Fradinho JC. 2024. The biotechnological potential of the Chloroflexota phylum. Appl Environ Microbiol 90:1–20. doi:10.1128/aem.01756-23 PMC11218635 38709098

[110] Ma Y, Wang J, Liu Y, Wang X, Zhang B, Zhang W, Chen T, Liu G, Xue L, Cui X. 2023. Nocardioides: “specialists” for hard-to-degrade pollutants in the environment. Molecules 28:1–18. doi:10.3390/molecules28217433 PMC10649934 37959852

[111] Sorouri B, Rodriguez CI, Gaut BS, Allison SD. 2023. Variation in Sphingomonas traits across habitats and phylogenetic clades. Front Microbiol 14:1–10. doi:10.3389/fmicb.2023.1146165 PMC10150699 37138640

[112] Shen Y, Ryser ET, Li H, Zhang W. 2021. Bacterial community assembly and antibiotic resistance genes in the lettuce-soil system upon antibiotic exposure. Sci Total Environ 778:1–9. doi:10.1016/j.scitotenv.2021.146255 33721642

[113] Brown LP, Murray R, Scott A, Tien YC, Lau CHF, Tai V, Topp E. 2022. Responses of the soil bacterial community, resistome, and mobilome to a decade of annual exposure to macrolide antibiotics. Appl Environ Microbiol 88:1–14. doi:10.1128/aem.00316-22 PMC9040571 35384705

[114] Romero F, Hilfiker S, Edlinger A, Held A, Hartman K, Labouyrie M, van der Heijden MGA. 2023. Soil microbial biodiversity promotes crop productivity and agro-ecosystem functioning in experimental microcosms. Sci Total Environ 885:1–10. doi:10.1016/j.scitotenv.2023.163683 37142020

[115] Delgado-Baquerizo M, Maestre FT, Reich PB, Jeffries TC, Gaitan JJ, Encinar D, Berdugo M, Campbell CD, Singh BK. 2016. Microbial diversity drives multifunctionality in terrestrial ecosystems. Nat Commun 7:1–8. doi:10.1038/ncomms10541 PMC4738359 26817514

[116] van Elsas JD, Chiurazzi M, Mallon CA, Elhottova D, Kristufek V, Salles JF. 2012. Microbial diversity determines the invasion of soil by a bacterial pathogen. Proc Natl Acad Sci USA 109:1159–1164. doi:10.1073/pnas.1109326109 22232669 PMC3268289

[117] Sah S, Krishnani S, Singh R. 2021. Pseudomonas mediated nutritional and growth promotional activities for sustainable food security. Curr Res Microb Sci 2:1–12. doi:10.1016/j.crmicr.2021.100084 PMC8645841 34917993

[118] Bull CT, De BS, Denny TP, Firrao G, Saux MF, Saddler GS, Scortichini M, Stead DE, Takikawa Y. 2010. Comprehensive list of names of plant pathogenic bacteria, 1980-2007. J Plant Pathol 92:551–592.

[119] Qin S, Xiao W, Zhou C, Pu Q, Deng X, Lan L, Liang H, Song X, Wu M. 2022. Pseudomonas aeruginosa: pathogenesis, virulence factors, antibiotic resistance, interaction with host, technology advances and emerging therapeutics. Signal Transduct Target Ther 7:1–27. doi:10.1038/s41392-022-01056-1 35752612 PMC9233671

[120] Brunhoferova H, Venditti S, Laczny CC, Lebrun L, Hansen J. 2022. Bioremediation of 27 micropollutants by symbiotic microorganisms of wetland macrophytes. Sustainability 14:1–16. doi:10.3390/su14073944

[121] Lei H, Zhang J, Huang J, Shen D, Li Y, Jiao R, Zhao R, Li X, Lin L, Li B. 2023. New insights into lincomycin biodegradation by Conexibacter sp. LD01: genomics characterization, biodegradation kinetics and pathways. J Hazard Mater 441:1–9. doi:10.1016/j.jhazmat.2022.129824 36087529

[122] Boss BL, Wanees AE, Zaslow SJ, Normile TG, Izquierdo JA. 2022. Comparative genomics of the plant-growth promoting bacterium Sphingobium sp. strain AEW4 isolated from the rhizosphere of the beachgrass Ammophila breviligulata. BMC Genomics 23:1–14. doi:10.1186/s12864-022-08738-8 35831788 PMC9281055

[123] Mahdi I, Fahsi N, Hijri M, Sobeh M. 2022. Antibiotic resistance in plant growth promoting bacteria: a comprehensive review and future perspectives to mitigate potential gene invasion risks. Front Microbiol 13:1–22. doi:10.3389/fmicb.2022.999988 PMC9530320 36204627

[124] Wash P, Batool A, Mulk S, Nazir S, Yasmin H, Mumtaz S, Alyemeni MN, Kaushik P, Hassan MN. 2022. Prevalence of antimicrobial resistance and respective genes among Bacillus spp., a versatile bio-fungicide. Int J Environ Res Public Health 19:1–14. doi:10.3390/ijerph192214997 PMC9690011 36429716

[125] Hou Q, Kolodkin-Gal I. 2020. Harvesting the complex pathways of antibiotic production and resistance of soil bacilli for optimizing plant microbiome. FEMS Microbiol Ecol 96:1–12. doi:10.1093/femsec/fiaa142 32672816

[126] Li X, Lu Z, Wu B, Xie H, Liu G. 2024. Antibiotics and antibiotic resistance genes removal in biological aerated filter. Bioresour Technol 395:1–12. doi:10.1016/j.biortech.2024.130392 38301943

[127] Tian S, You L, Huang X, Liu C, Su JQ. 2024. Efficient sulfamethoxazole biotransformation and detoxification by newly isolated strain Hydrogenophaga sp. SNF1 via a ring ortho-hydroxylation pathway. J Hazard Mater 480:1–11. doi:10.1016/j.jhazmat.2024.136113 39405676

[128] Zhang Y, Zheng X, Xu X, Cao L, Zhang H, Zhang H, Li S, Zhang J, Bai N, Lv W, Cao X. 2022. Straw return promoted the simultaneous elimination of sulfamethoxazole and related antibiotic resistance genes in the paddy soil. Sci Total Environ 806:1–10. doi:10.1016/j.scitotenv.2021.150525 34582855

[129] Ajibade FO, Yin WX, Guadie A, Ajibade TF, Liu Y, Kumwimba MN, Liu WZ, Han JL, Wang HC, Wang AJ. 2023. Impact of biochar amendment on antibiotic removal and ARGs accumulation in constructed wetlands for low C/N wastewater treatment. Chem Eng J 459:1–11. doi:10.1016/j.cej.2023.141541

[130] Li T, Cao X, Wu Z, Liu J, Hu B, Chen H, Li B. 2023. Biotransformation of nitrogen and tetracycline by counter-diffusion biofilm system: multiple metabolic pathways, mechanism, and slower resistance genes enrichment. Chem Eng J 474:1–15. doi:10.1016/j.cej.2023.145637

[131] Garbini GL, Grenni P, Rauseo J, Patrolecco L, Pescatore T, Spataro F, Barra Caracciolo A. 2022. Insights into structure and functioning of a soil microbial community amended with cattle manure digestate and sulfamethoxazole. J Soils Sediments 22:2158–2173. doi:10.1007/s11368-022-03222-y

[132] Jiao S, Chen W, Wei G. 2019. Resilience and assemblage of soil microbiome in response to chemical contamination combined with plant growth. Appl Environ Microbiol 85:1–16. doi:10.1128/AEM.02523-18 PMC6414375 30658982

[133] Thiele‐Bruhn S. 2003. Pharmaceutical antibiotic compounds in soils – a review. J Plant Nutr Soil Sci 166:145–167. doi:10.1002/jpln.200390023

[134] Vives-Peris V, de Ollas C, Gómez-Cadenas A, Pérez-Clemente RM. 2020. Root exudates: from plant to rhizosphere and beyond. Plant Cell Rep 39:3–17. doi:10.1007/s00299-019-02447-5 31346716

[135] Keenum I, Liguori K, Calarco J, Davis BC, Milligan E, Harwood VJ, Pruden A. 2022. A framework for standardized qPCR-targets and protocols for quantifying antibiotic resistance in surface water, recycled water and wastewater. Crit Rev Environ Sci Technol 52:4395–4419. doi:10.1080/10643389.2021.2024739

[136] Osose EB, Okafor COO, Nnenna OE, Ndidi OK, Ikechukwu O, Francisca AU, Felicia ON, Chinyere NA, Romanus II. 2025. Multidrug resistant Salmonella species harboring sulfonamide (sul_1_ and sul_2_) resistant gene isolated from typhoid patients in a university teaching hospital – A threat to public health. Total Environ Microbiol 1:1–7. doi:10.1016/j.temicr.2025.100021

[137] Moja L, Zanichelli V, Mertz D, Gandra S, Cappello B, Cooke GS, Chuki P, Harbarth S, Pulcini C, Mendelson M, et al.. 2024. WHO’s essential medicines and AWaRe: recommendations on first- and second-choice antibiotics for empiric treatment of clinical infections. Clin Microbiol Infect 30:S1–S51. doi:10.1016/j.cmi.2024.02.003 38342438

[138] Yin L, Wang X, Li Y, Liu Z, Mei Q, Chen Z. 2023. Uptake of the plant agriculture-used antibiotics oxytetracycline and streptomycin by cherry radish─effect on plant microbiome and the potential health risk. J Agric Food Chem 71:4561–4570. doi:10.1021/acs.jafc.3c01052 36945880

[139] Cui M, Yu S, Yu Y, Chen X, Li J. 2022. Responses of cherry radish to different types of microplastics in the presence of oxytetracycline. Plant Physiol Biochem 191:1–9. doi:10.1016/j.plaphy.2022.09.012 36162140

[140] Jia WL, Gao FZ, Song C, Chen CE, Ma CX, White JC, Ying GG. 2024. Swine wastewater co-exposed with veterinary antibiotics enhanced the antibiotic resistance of endophytes in radish (Raphanus sativus L.). Environ Pollut 362:1–13. doi:10.1016/j.envpol.2024.125040 39343351

[141] Chung HS, Lee YJ, Rahman MM, Abd El-Aty AM, Lee HS, Kabir MH, Kim SW, Park BJ, Kim JE, Hacımüftüoğlu F, Nahar N, Shin HC, Shim JH. 2017. Uptake of the veterinary antibiotics chlortetracycline, enrofloxacin, and sulphathiazole from soil by radish. Sci Total Environ 605–606:322–331. doi:10.1016/j.scitotenv.2017.06.231 28668743

[142] Sidhu H, O’Connor G, Kruse J. 2019. Plant toxicity and accumulation of biosolids-borne ciprofloxacin and azithromycin. Sci Total Environ 648:1219–1226. doi:10.1016/j.scitotenv.2018.08.218 30340267

[143] Nafees M, Qiu L, Alomrani SO, Ahmad Z, Yin Y, Sallah A AH, Alshehri MA, Ali S, Guo H. 2025. Enhancing spinach growth and soil microbial health under sulfadiazine and polypropylene exposure through zinc fortification. Environ Technol Innov 38:1–22. doi:10.1016/j.eti.2025.104186

[144] Mohy-u-Din N, Farhan M, Wahid A, Ciric L, Sharif F. 2023. Human health risk estimation of antibiotics transferred from wastewater and soil to crops. Environ Sci Pollut Res 30:20601–20614. doi:10.1007/s11356-022-23412-y 36255570

[145] Khan NM, Imran M, Ashraf M, Arshad H, Awan AR. 2022. Oxytetracycline and ciprofloxacin antibiotics exhibit contrasting effects on soil microflora, nitrogen uptake, growth, and yield of wheat (Triticum aestivum L.). J Soil Sci Plant Nutr 22:3788–3797. doi:10.1007/s42729-022-00927-4

[146] Liu F, Ying GG, Tao R, Zhao JL, Yang JF, Zhao LF. 2009. Effects of six selected antibiotics on plant growth and soil microbial and enzymatic activities. Environ Pollut 157:1636–1642. doi:10.1016/j.envpol.2008.12.021 19157661

[147] Timmerer U, Lehmann L, Schnug E, Bloem E. 2020. Toxic effects of single antibiotics and antibiotics in combination on germination and growth of Sinapis alba L. Plants (Basel) 9:1–19. doi:10.3390/plants9010107 PMC7020151 31952171

