# Supplementary material for: Soil microbial and plant responses to increasing antibiotic concentration: a case study of five antibiotics
Source: Appl Environ Microbiol. 2026 Feb 24;92(3):e01581-25. doi: 10.1128/aem.01581-25 (PMC12997804; doi:10.1128/aem.01581-25)
Supplement: Supplemental material — Supplemental text, Tables S1 to S9, and Fig. S1 to S5. [file aem.01581-25-s0001.docx]

**Appendix to Soil microbial and plant responses to increasing antibiotic concentration: a case study of five antibiotics**

Sarah van den Broek^1,2*^, Inna Nybom^1,3^, Rafaela Feola Conz^2^, Yifei Sun^1,4^, Thomas D. Bucheli^3^, Sebastian Doetterl^1^, Martin Hartmann^2^, Gina Garland^1,5^

^1^ Soil Resources, Institute of Terrestrial Ecosystems, Department of Environmental Systems Science, ETH Zürich, Switzerland

^2^ Sustainable Agroecosystems, Institute of Agricultural Sciences, Department of Environmental Systems Science, ETH Zürich, Switzerland

^3^ Environmental Analytics, Agroscope Reckenholz, Switzerland

^4^ Institute of Environment and Sustainable Development in Agriculture, Chinese Academy of Agricultural Sciences/Key Laboratory of Agro-Environment, Ministry of Agriculture, China

^5^ Soil Quality and Soil Use, Agroscope Reckenholz, Switzerland

*Corresponding author. E-mail address: [sarah.vandenbroek@usys.ethz.ch](mailto:sarah.vandenbroek@usys.ethz.ch)

**Content**

[Supplementary Text sections 2](#_Toc217406698)

[Text section 1: Antibiotic extraction from bulk sand-soil mixture 2](#_Toc217406699)

[Text section 2: Antibiotic instrumental analysis 2](#_Toc217406700)

[Text section 3: QC/QA of antibiotic analysis 3](#_Toc217406701)

[Text section 4: Spike stock analysis and initial measured soil concentrations 4](#_Toc217406702)

[Text section 5: PCR protocol for 16S and ITS 4](#_Toc217406703)

[Text section 6: PCR protocol for ARGs and MGEs 5](#_Toc217406704)

[Supplementary Tables 6](#_Toc217406705)

[Table S1 Soil properties of the starting soil. 6](#_Toc217406706)

[Table S2 Antibiotic standards and chemicals used in antibiotics extraction 7](#_Toc217406707)

[Table S3 Physicochemical characteristics of the antibiotics and chemical structures. 8](#_Toc217406708)

[Table S4 Applied antibiotic concentrations. 9](#_Toc217406709)

[Table S5 Details on analytical method of antibiotic analysis, instrument and method precision 10](#_Toc217406710)

[Table S6 Primers used in this study 11](#_Toc217406711)

[Table S7 Prokaryotic and fungal alpha diversity 12](#_Toc217406712)

[Table S8 Prokaryotic and fungal community beta diversity. 12](#_Toc217406713)

[Table S9 Soil and plant parameters. 13](#_Toc217406714)

[Supplementary Figures 14](#_Toc217406715)

[Figure S1 PCoA Unconstrained analysis of prokaryotic and fungal communities 14](#_Toc217406716)

[Figure S2 Prokaryotic genera known for antibiotic-related mechanisms. 15](#_Toc217406717)

[Figure S3 In silico PCR results 16](#_Toc217406718)

[Figure S4 IntI1 and sul1 genes 17](#_Toc217406719)

[Figure S5 Soil pH 18](#_Toc217406720)

[References 19](#_Toc217406721)

## Supplementary Text sections

### Text section 1: Antibiotic extraction from bulk sand-soil mixture

The total antibiotic concentration of the bulk sand-soil mixture was determined following the method described in Shi et al. (2022). For extraction the samples, 2.5g c0, c0.1, c1.0 or 1.25g c10 wet weight, was weighed in 50ml screwcap centrifuge tubes (polypropylene) and 100 µl of internal standard solution was added. The internal standard stock solution consisted isotopically labeled internal standards Clarithromycin-N-methyl-13C,d3 (CLR-13C-D3), Sulfamethoxazole-d4 (SMX-D4) and Trimethoprim-d3 (TMP-D3), as specified in Table S2. 80 ng (c0, c0.1, c1) or 400 ng (c10) of internal standard were added to the samples (stock solution concentrations of 0.8 ng µl^-1^ and 4 ng µl^-1^ respectively) leading to 20 ng ml^-1^ final concentration in the samples. Following the addition of the internal standard stock solution the samples were left standing for one hour prior to extraction. Due to the high analyte concentration in samples from c10, a lower sample mass was used, and the final sample extract was diluted (× 10) prior to analysis. To extract the samples, EDTA disodium dihydrate (0.4 g) was added, followed by the extraction solvents, 10 mL of acetonitrile (acidified with 5% formic acid) and 10 mL of potassium phosphate buffer (pH 3). The sample was vortexed (3 min) (OHAUS Multi-Tube Vortexer, VXMTALB) and centrifuged (4000 rpm, 5 min) (3578 RCF, Hettich, Rotanta 460R). The supernatant was decanted to a new 50 ml screwcap centrifuge tube. The extraction of the sample was repeated for a second time as described above and the supernatant was pooled. Sodium chloride (10 g) was added to the pooled supernatants for phase separation, the centrifuge tube was shaken by hand for 15 seconds and centrifuged (4000 rpm, 5 min). From the solvent phase (top), 1.5 ml sample was collected and placed in a 2 ml amber glass screw-thread vial and 75 µl ammonia (7 Molar) was added to buffer the low pH of the sample during solvent change. The sample was evaporated to dryness under a gentle stream of nitrogen flow and re-constructed to 300 µl water:methanol (70%:30%, v;v), acidified with 1% formic acid, and centrifuged (4000 rpm, 5 min) before analysis. Further sample clean-up was omitted.

### Text section 2: Antibiotic instrumental analysis

The instrumental analysis of the antibiotics was conducted directly after extraction to avoid potential losses of analytes during storage. Samples were analyzed on an Agilent liquid chromatography-triple quadrupole mass spectrometry system (LC-MS/MS, 6470, Agilent Technologies). The analytes were separated on a C18 column (Agilent ZORBAX RR Eclipse Plus 95Å, 3.0 × 100 mm, particle size 3.5 µm) at 40 ⁰C (Agilent Column Oven 1260 Infinity II, Santa Clara, USA), and 5 μL of sample was injected (Agilent Autosampler 1260 Infinity II, Santa Clara USA). Ultrapure water (Phase A) and methanol (Phase B) with 0.1% formic acid were used as mobile phases at a flow rate of 0.4 ml min^-1^(Agilent Pump 1290 Infinity I, Santa Clara, USA). Phase B was linearly increased from 2% to 95% over the course of 12 min, held constant for 4 min, after which the phase B was returned to 2% and held constant for 3 min prior to the next sample injection. The mass spectrometer was operated with electrospray ionization in positive mode. For each target analyte and internal standard three multiple reaction monitoring transitions were used including one quantifier and two qualifiers (Table S5).

A matrix matched calibration was used for quantification of the samples. For this, an additional matrix sample of the control soil without addition of antibiotics was processed and extracted as described above for the samples. Ten aliquots from the matrix extract were collected and evaporated with nitrogen flow as described above for the samples. Calibration samples were prepared in these aliquots by adding water:methanol ratio of 70%:30%, v;v and acidified with 1% formic acid and analyte concentrations of 0, 0.1, 0.5, 1, 5, 10, 50, 100 and 500 ng ml^-1^. Isotopically labeled internal standards (CLR-13C-D3, SMX-D4 and TMP-D3) were added to each calibration level at a concentration of 20 ng ml^-1^ before analysis. One additional matrix sample was used as a laboratory and analytical blank without addition of analytes or internal standards. For analytes without a structural identical internal standard available (CTC, ENR), the quantification was done with matrix matched calibration and the calculated concentrations were corrected with the pre-determined absolute recovery. Absolute recoveries were determined to identify analyte losses during the extraction process. For this, additional samples from the control soil were spiked with analytes in triplicate at three concentration levels (5, 50 and 500 ng g^-1^ DW). The samples were left standing for one hour and extracted as previously described. Directly prior to analysis the structurally identical isotope labelled internal standards were added to the recovery sample. The percentage of recovery was calculated from the peak area ratios of the analytes in the extract compared to the peak area ratios in the matrix matched standards with equivalent concentration levels. The absolute recoveries are presented in Table S4.

### Text section 3: QC/QA of antibiotic analysis

The limit of detection (LOD) and limit of quantification (LOQ) were determined from the signal-to-noise ratio, where the LOD was defined as the lowest analyte concentration at which the S/N was at least 3:1, and for LOQ S/N ratio of at least 10:1. The determined LOQs were ≤ 3.19 µg kg^-1^ DW with highest LOQ observed with CTC (Table S4). To determine the analytical method repeatability, one or two samples in each analysis batch were injected in triplicate. Extraction method reproducibility and robustness was evaluated by including an additional spiked control soil sample to each extraction batch, where the control soil was spiked prior to extraction with 100 µl analyte standard solution and 100 µl of internal standard solution (CLR-13C-D4, SMX-D4 and TMP-D3) at 0.8 ng µl^-1^ each, leading to 20 ng ml^1^ final concentration of internal standards and analytes in the samples. For sampling representativeness, two selected pots from each treatment were sampled and extracted in triplicate (variation within sample). The instrument and method precision were assessed by calculating coefficient of variation (CV %):

$$\text{CV (\%) = }\left( \frac{Standard deviation}{Mean} \right) \times100$$

The instrument precision proved to be highly reproducible (CV ≤ 3.3%). Variation within the samples proved to be at the same level compared to the variation between analytical replicates, thus demonstrating that the sampling was representative and the soil spiking resulted in homogeneous exposure concentrations. The highest variation of extracted concentrations was observed in analysis of CTC (CV 17.3 %). In addition, between-batch CV of the spiked samples were 20.7% for CTC and 17.6% for SMZ (Table S4). The variation of c10 treatment was assessed separately, since smaller sample mass was used for the analysis (1g DW). The variation within sample and between replicates was found to be at the same range in c0.1 and c1 treatments compared to the c10 treatment, thus also confirming that the samples were representative despite the smaller sample mass (Table S4).

### Text section 4: Spike stock analysis and initial measured soil concentrations

The stock solutions used for spiking were analyzed to calculate the expected antibiotic concentration in the soil. The analytical method described above for samples (SI Text section 3) was used to analyze the samples. However, due to the high concentrations of the stock solutions (0.1; 1; and 10 mg ml^-1^ SMZ, TMP, CLA and CTC and 0.04; 0,4; and 4 mg ml^-1^ ENR) dilutions up to 5 orders of magnitude were required prior to analytical measurement. Therefore, the determined concentrations should be considered as indicative rather than absolute. The calculated applied concentrations based on the analyzed stock solution concentrations were well in line with the intended spike levels, excluding CLR c0.1 treatment (Table S4). For the CLR c0.1 stock solution preparation an error was made leading to an order of magnitude lower concentration than intended. As the concentrations of the stock solutions were only analyzed after the start of the experiment the error was noted too late for correction. The lower stock solution concentrations of c0.1 CLR was also reflected in the determined soil concentrations as shown in Table 1.

The CLR D0 soil concentrations were 0.014 mg kg^-1^ c0.1 and for c1 and c10 concentrations were close to the spiking level namely 1.09 and 11.33 mg kg^-1^ respectively (Table 1). The D0 soil concentrations of ENR, SMX, and CTC were lower than expected based on the spiked concentrations (Table 1 and Table S4). For example, the determined concentrations in c10 soil were 8.4, 6.8 and 5.2 mg kg^-1^ respectively. The lower D0 soil concentrations of ENR, SMX, and CTC can potentially be explained by adsorption to wall of the pots or strong sorption to soil-forming non-extractable residues. Some immediate degradation may have also taken place, given that after spiking the soil-sand mixtures were left to evaporate in room temperature as described in materials and methods (section 2.1), and the DO samples were only collected and stored in freezer (-20 °C) after the pots were established. Volatilization of the antibiotics is not expected to play a major role in their dissipation. For example, the Henry’s law constants for antibiotics are very low to negligible (e.g. for sulfonamides 1.3 × 10^-15^ - 1.8 × 10^-11^ Pa m³mol⁻¹) (2).

### Text section 5: PCR protocol for 16S and ITS

The PCR mixture consisted of GoTaq Colorless Mastermix 1x (Promega, Madison, WI, United States), 0.5 mM MgCl_2_ and 1.0 mM MgCl_2_ for 16S and ITS respectively, 0.4 μM forward primer, 0.4 μM reverse primer and 40 ng template to a final volume of 25 μl, pipetted with QIAgility (Qiagen, Hilden, Germany). Amplification PCR program followed the protocol: initial denaturation (2 min, 95 °C), denaturation (40 s, 95 °C), annealing (40 s, 58°C), extension (1 min, 72 °C) and final extension (10 min, 72 °C) for 30 cycles to amplify 16S and 35 cycles to amplify ITS performed with C1000^TM^ Touch Thermal Cycler (Bio-Rad Laboratories, Hercules, CA, United States). PCR amplification was tested with and without bovine serum albumin (BSA) on a few samples representing a range of DNA and purity levels which did not yield different amplification results on the gel electrophoresis using the QIAxcel System (Qiagen). Therefore, the PCR amplification for all samples was conducted without BSA. PCR amplification was conducted using three technical replicates. The PCR samples were then analyzed with gel electrophoresis using the QIAxcel System (Qiagen) to ensure successful amplification and evenness of technical replicates.

### Text section 6: PCR protocol for ARGs and MGEs

DNA extracts were pooled by the four different antibiotic treatments and normalized to 10 ng μl^-1^. Then, a temperature gradient PCR (95 °C denaturation, 53.6-63.6 °C annealing, 72 °C extension, 38 cycles) was conducted using the BioRad C1000^TM^ Touch Thermal Cycler (Bio-Rad Laboratories) to find the ideal annealing temperature for the specific primer set. The PCR reactions were conducted using GoTaq Colorless Mastermix 1x, 0.4 µM forward primer, 0.4 µM reverse primer and 40 ng template to a final volume of 25 µl pipetted with QIAgility. The results were analyzed with gel electrophoresis using the QIAxcel System (Qiagen, Hilden, Germany). No amplification of *tetQ*, *intI1*, *dfrA12* and *qnrS1* was found and an additional temperature gradient was done for *tetQ* (45.0 – 53.6 °C) and *qnrS1* (63.0 – 68.0 °C) to ensure the correct annealing temperature was not missed. Only *sul1* and *intI1* had successful amplification and qPCR was subsequently conducted to perform a quantitative analysis of these two genes. For qPCR, the standard curve was made by purifying the PCR products with the E.Z.N.A. Cycle Pure V-spin kit (Omega Bio-tek, Georgia, USA) and quantities were determined with the QIAxpert System (Qiagen). Before qPCR reactions were conducted, potential amplification inhibition was tested across all samples by spiking pGEM-T plasmid (GenBank® Accession No. X65308; Promega, Madison, WI, USA) into the soil DNA at equimolar concentrations in all samples and amplifying a region on the plasmid using specific primers SP6 and T7 (Microsynth, Balgach, Switzerland). As no amplification inhibition was detected, touchdown qPCR was conducted using the CFX97 Real Time PCR Thermal Cycler (Bio-Rad Laboratories) to avoid unspecific amplification. For both *sul1* and *intI1* the following program was used: initial denaturation (3 min, 95 °C), 6 cycles touchdown qPCR: denaturation (15 s, 95 °C), annealing and extension (1 min, 65 °C, -1 °C per cycle), fluorescence detection (15 s, 80 °C), followed by 24 and 32 cycles for *sul1* and *intI1* respectively of denaturation (15 s, 95 °C), annealing and extension (1 min, 60 °C), fluorescence detection (15 s, 80 °C) and final melt curve (from 65 °C to 95 °C, decrease of 0.5 °C every 30 s) for a total of 30 and 38 cycles respectively. For qPCR of 16S, the following qPCR program was used for 30 cycles: initial denaturation (3 min, 95 °C), denaturation (30 s, 95 °C), annealing (40 s, 52 °C), extension (30 s, 72 °C), fluorescence detection (15 s, 80 °C) and final melt curve (from 65 °C to 95 decrease of 0.5 °C every 30 s). The amplification efficiencies ranged from 90.6 to 101.7% and the coefficients of determination (R^2^) ranged from 0.999 to 1.000 across the technical replicates for *sul1*, 82.2 to 86.5% with an R^2^ of 1.000 for *intI1* and 87.5 to 88.6% with an R^2^ of 1.000 for 16S. The qPCR reactions were conducted using Sso Advanced™ Universal SYBR® Green Supermix (Bio-Rad Laboratories) 0.25 μM forward and reverse primer for *sul1* and *int1* and 0.8 μM of primers forward and reverse for 16S, and 40 ng of DNA template.

# Supplementary Tables

### Table S1 Soil properties of the starting soil. ^1^Based on the World Reference Base for Soil Resources. ^2^ Reactive pedogenic oxides based on the sum of pyrophosphate and ammonium oxalate extractable fractions.

| **Soil property** | **Mean ± SD** |
| --- | --- |
| pH | 7.36 ± 0.06 |
| Clay (%)^1^ | 6.2 |
| Silt (%)^1^ | 44.1 |
| Sand (%)^1^ | 49.7 |
| NO_3_^-^-N (mg kg^-1^) | 49.20 ± 6.65 |
| NH_4_^+^-N (mg kg^-1^) | 0.0291 ± 0.0362 |
| P (mg kg^-1^) | 107.0 ± 10.5 |
| K (mg kg^-1^) | 104.0 ± 14.8 |
| Ca (mg kg^-1^) | 8891 ± 638 |
| Cu (mg kg^-1^) | 9.080 ± 0.663 |
| Mg (mg kg^-1^) | 182.0 ± 18.9 |
| Mn (mg kg^-1^) | 153.0 ± 20.1 |
| S (mg kg^-1^) | 85.9 ± 10.6 |
| Zn (mg kg^-1^) | 18.10 ± 3.47 |
| NO_3_^-^-N (mg l^-1^) | 12.3 ± 1.64 |
| NH_4_^+^-N (mg l^-1^) | 0.00727 ± 0.00905 |
| P (mg l^-1^) | 8.600 ± 0.624 |
| K (mg l^-1^) | 2.93 ± 0.07 |
| Mg (mg l^-1^) | 8.4 ± 0.2 |
| Al (mg kg^-1^)^2^ | 1257 ± 6.8 |
| Fe (mg kg^-1^)^2^ | 1618 ± 2.2 |
| Mn (mg kg^-1^)^2^ | 231 ± 1.3 |
| Si (mg kg^-1^)^2^ | 1589 ± 9.4 |

### Table S2 Antibiotic standards and chemicals used in antibiotics extraction

|  | **Cas** | **Assay** | **Supplier** |
| --- | --- | --- | --- |
| **Antibiotics and analytical standards** | | | |
| Chlortetracycline -HCL (CTC) | 64-72-2 | 90.9% | Supelco |
| Enrofloxacin (ENR) | 93106-60-6 | 99.7% | Supelco |
| Clarithromycin (CLR) | 81103-11-9 | 98.7% | Supelco |
| Sulfamethoxazole (SMX) | 723-46-6 | 99.8% | Sigma-Aldrich |
| Trimethoprim (TMP) | 723-46-6 | 99.8% | Supelco |
| Clarithromycin-N-methyl-13C,d3* (CLR-13C-D3) | NA | 96.0% | TRC Canada |
| Sulfamethoxazole -d4* (SMX-D4) | 1020719-86-1 | 98.0% | TRC Canada |
| Trimethoprim -d3* (TMP-D3) | 1189923-38-3 | 97.0% | TRC Canada |
| **Antibiotic extraction and analytics** | | | |
| Acetonitrile | 75-05-8 | ≥99.9% | VWR |
| Methanol | 67-56-1 | ≥99.9% | VWR |
| Water | 7732-18-5 |  | VWR |
| Formic acid | 64-18-6 | 99.6% | VWR |
| EDTA disodium dihydrate (Na_2_EDTA·2H_2_O) | 6381-92-6 | 100.3% | VWR |
| Potassium phosphate monobasic ^a^ | 7778-77-0 | 99.50% | Fluka |
| Ortho-Phosphoric acid 85%^a^ | 7664-38-2 | >85% | Fluka |
| Sodium Chloride | 7647-14-5 | ≥99.5% | Merck |
| Ammonia, 7M in Methanol | 7664-71-7 |  | thermo scientific |
|  |  |  |  |
| ^a^ Potassium phosphate buffer (pH3) was prepared form potassium dihydrogen phosphate (27.2g) and water (1L) and acidified with ortho-phosphoric acid (1.35ml) to pH 3 | | | |

### Table S3 Physicochemical characteristics of the antibiotics and chemical structures. Data from (3–11).

|  |  |
| --- | --- |
| **Chlortetracycline (CTC)** | Formula: C_22_H_23_ClN_2_O_8_ |
| 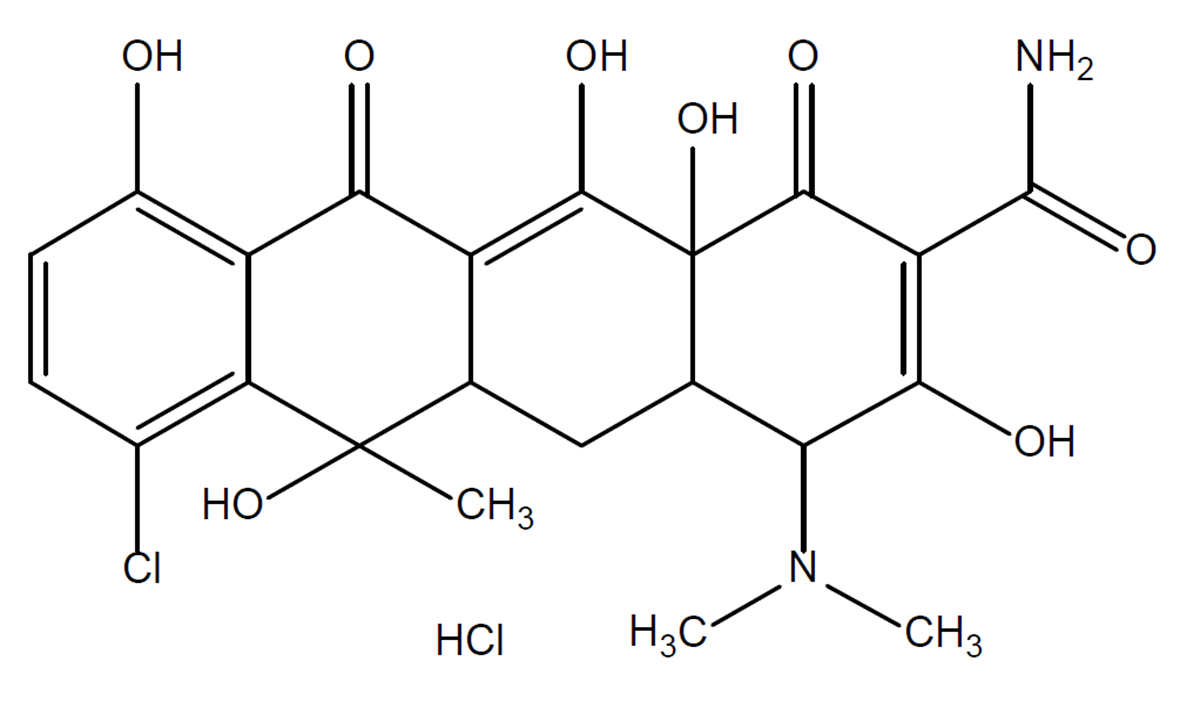 | Molecular weight: 515.3 g mol^-1^ |
|  | Water solubility: 0.6 g L^-1^ |
|  | pKa1: 3.30 Pka2: 7.44 Pka3: 9.27 |
|  | Log Koc: 2.9 L Kg^-1^ |
| **Enrofloxacin (ENR)** | Formula: C_19_H_22_FN_3_O_3_ |
| 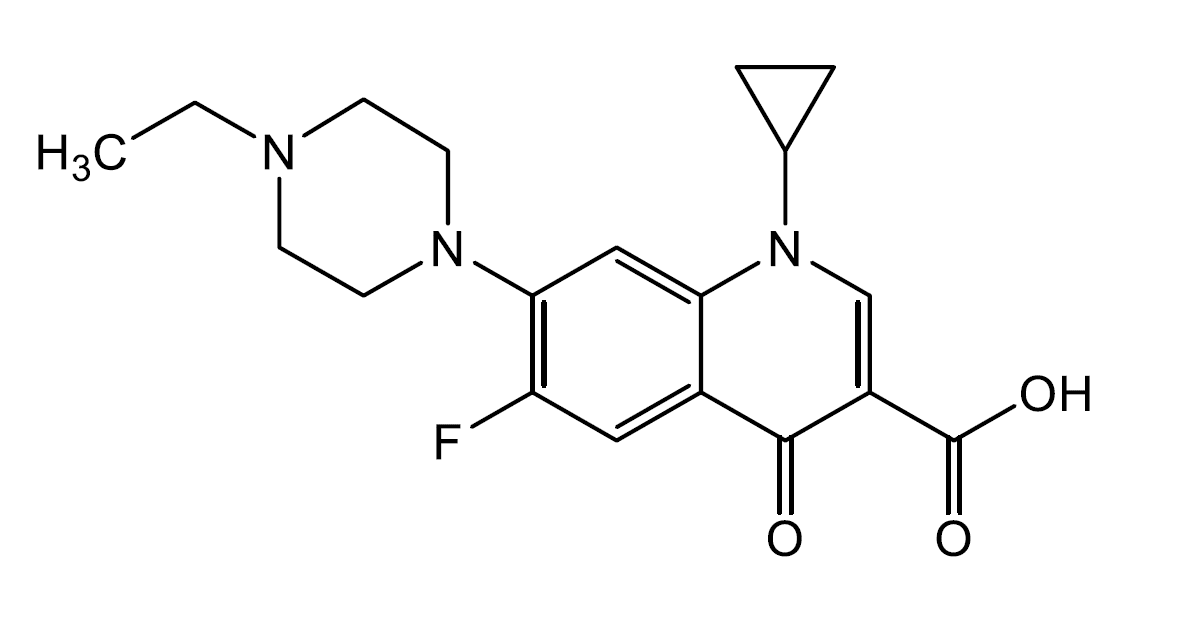 | Molecular weight: 359.39 g mol^-1^ |
|  | Water solubility: 130 g L^-1^ |
|  | pKa1: 6.0 pKa2: 8.0 |
|  | Log Koc: 4.2-5.9 L Kg^-1^ |
| **Clarithromycin (CLR)** | Formula: C_38_H_69_NO_13_ |
| 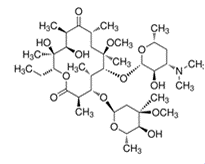 | Molecular weight: 747.95 g mol^-1^ |
|  | Water solubility: 0.0017 g/L |
|  | pKa1: 8.99 |
|  | Log Koc: 2.2 L Kg^-1^ |
| **Sulfamethoxazole (SMX)** | Formula: C_10_H_11_N_3_O_3_S |
| 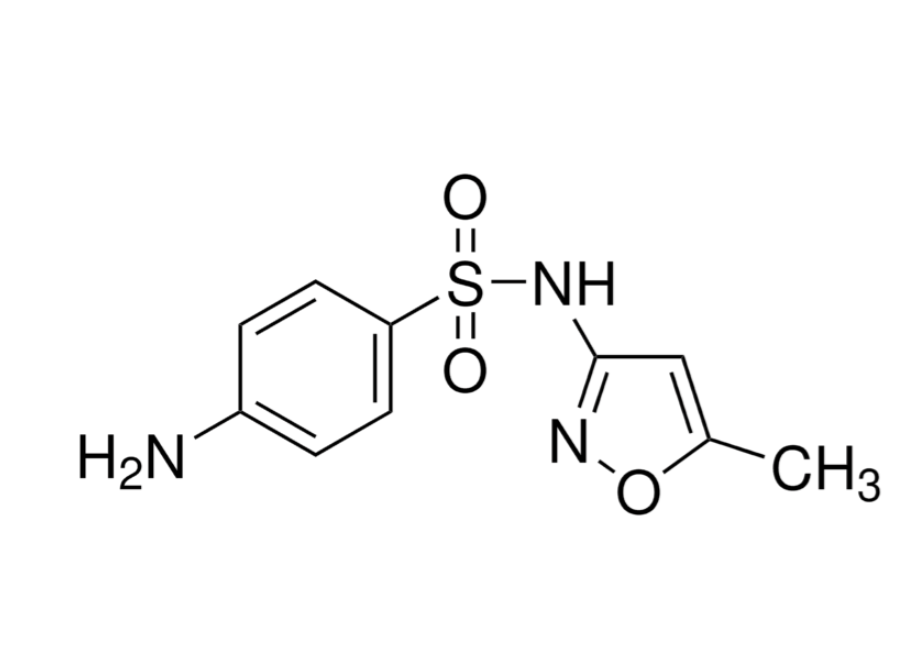 | Molecular weight: 253.28 g mol^-1^ |
|  | Water solubility: 0.61 g L^-1^ |
|  | pKa1: 1.39, pKa2: 5.8 |
|  | Log Koc: 2.0 |
| **Trimethoprim (TMP)** | Formula: C_14_H_18_N_4_O_3_ |
| 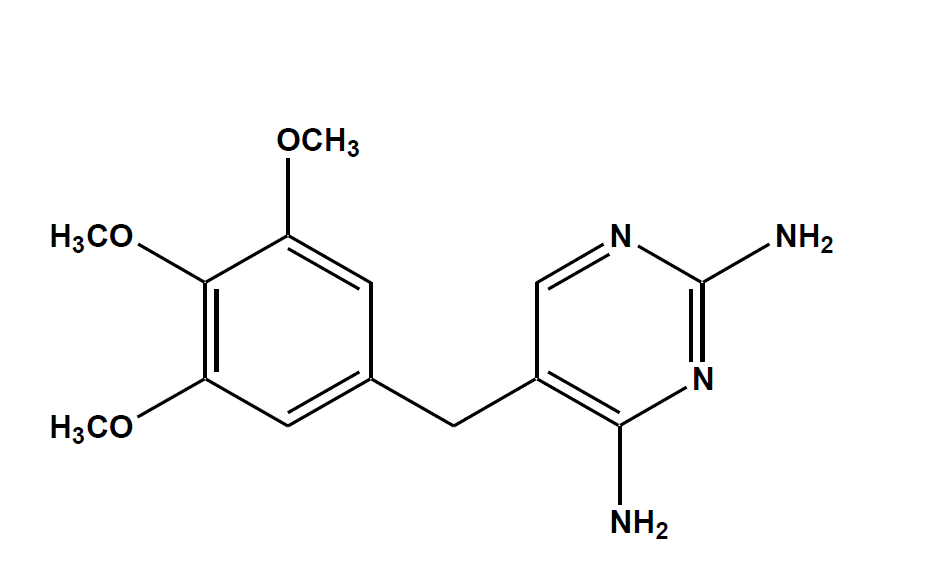 | Molecular weight: 290.3 g mol^-1^ |
|  | Water solubility: 0.4 g L^-1^ |
|  | pKa1: 7.2 |
|  | Log Koc: 3.7 |
| Data from Sarmah et al 2006, Stephens et al 1956, Cycoń et al 2019 (CTC); Nowara et al 1997, Boxall et al 2006 (ENR); Cycoń et al 2019, McFarland et al 1997 (CLA); Lin and Gan 2011, Avisar et al 2010, Stoob et al 2007 (SMZ); Lin and Gan 2011, Boxall et al 2006 (TMP) | |

### Table S4 Applied antibiotic concentrations. Target antibiotic concentration (antibiotic treatment) and nominal applied antibiotic concentration based on measured stock solution concentrations.

| **Antibiotic treatment**  *mg kg^-1^* | **Applied antibiotic concentration^a^**  *mg kg^-1^* |
| --- | --- |
| **Chlortetracycline (CTC)** | |
| 0 | - |
| 0.1 | 0.2 |
| 1 | 2.3 |
| 10 | 14.5 |
| **Clarithromycin (CLR)** | |
| 0 | - |
| 0.1 | 0.01 |
| 1 | 1.2 |
| 10 | 11.5 |
| **Enrofloxacin (ENR)** | |
| 0 | - |
| 0.1 | 0.2 |
| 1 | 0.9 |
| 10 | 12.5 |
| **Sulfamethoxazole (SMX)** | |
| 0 | - |
| 0.1 | 0.1 |
| 1 | 1.1 |
| 10 | 11.6 |
| **Trimethoprim (TMP)** | |
| 0 | - |
| 0.1 | 0.1 |
| 1 | 1.3 |
| 10 | 12.1 |

### Table S5 Details on analytical method of antibiotic analysis, instrument and method precision and limits of detection (LOD) and quantification (LOQ).

| Class | Tetracyclines | Fluoroquinolones | Sulfonamides | Diaminopyrimidines | Macrolides |
| --- | --- | --- | --- | --- | --- |
| Name | Chlorotetracycline | Enrofloxacin | Sulfamethoxazole | Trimethoprim | Clarithromycin |
|  | (CTC) | (ENR) | (SMX) | (TMP) | (CLR) |
| RT (min) | 7.37 | 6.10 | 6.29 | 5.08 | 11.13 |
| ionization mode | pos | pos | pos | pos | pos |
| MRM_quant_ | 444 | 342.2 | 156 | 230.1 | 158.1 |
| MRM_qual 1_ | 154.1 | 316.2 | 92.1 | 261.1 | 590.4 |
| MRM_qual 2_ | 98.1 | 245.1 | 65.1 | 123.1 | 83 |
| ISTD* MRM_quant_ | - | - | 160 | 230.1 | 162 |
| ISTD* MRM_qual 1_ | - | - | 112 | 264.1 | 594.4 |
| ISTD* MRM_qual 2_ | - | - | 96.1 | 123.1 | 83.1 |
| Linear range (ng/ml) | 1-100 | 0.1-100 | 0.1-500 | 0.1-500 | 0.1-500 |
| lowes cal. level (ng/ml) | 1 | 0.1 | 0.1 | 0.1 | 0.1 |
| R2 | 0.976 | 0.975 | 0.999 | 0.997 | 0.998 |
| Reproducibility of response, CV% | 3.3 | 1.1 | 1.9 | 0.8 | 1.1 |
| CV (%) within sample,  treatments 0.1 and 1 mg/kg | 17.3 | 9.1 | 14.5 | 14.8 | 8.4 |
| CV (%) within sample, treatment 10 mg/kg | 10.1 | 9.2 | 15.0 | 12.6 | 6.6 |
| CV (%) within replicates,  treatments 0.1 and 1 mg/kg | 7.0 | 8.1 | 14.9 | 14.9 | 6.3 |
| CV (%) within replicates,  treatment 10 mg/kg | 4.7 | 6.4 | 15.7 | 11.0 | 6.3 |
| CV (%) day-to-day,  spiked sample | 20.7 | 7.4 | 17.6 | 15.0 | 12.1 |
| Absolute recovery % | 87.3 ± 8.4 | 86.2 ± 6.5 | 77.5 ± 7.3 | 89.3 ± 5.0 | 104.8 ± 5.9 |
| LOD (ng/g dw) | 0.96 | 0.09 | 0.32 | 0.01 | 0.13 |
| LOQ (ng/g dw) | 3.19 | 0.29 | 1.08 | 0.03 | 0.43 |

*ISTD = structural identical isotope labelled internal standards SMX-D4, TMP-D3 and CLR-13C-D3

### Table S6 Primers used in this study

| **Gene** | **Forward primer** | **Reverse primer** | **Ref** |
| --- | --- | --- | --- |
| 16S rRNA gene V4 region | 341F (5’-CCTAYGGGDBGCWSCAG-3’) | 806R (5’-GGACTACNVGGGTHTCTAAT-3’) | (12) |
| ITS region | ITS3ngs (5’-CANCGATGAAGAACGYRG-3’) | ITS4ngs (5’-CCTSCSCTTANTDATATGC-3’) | (13) |
| TRUESEQ Sequencing tags | 5’- CTTTCCCTACACGACGCTCTTCCGATCT-3’ | 5’- GGAGTTCAGACGTGTGCTCTTCCGATCT-3’ |  |

| **Gene** | **Resistance to antibiotic in this study** | **Drug class** | **Mechanism** | **Forward primer** | **Reverse primer** | **Amplicon length (bp)** | **Annealing temperature (°C)** | **Ref** |
| --- | --- | --- | --- | --- | --- | --- | --- | --- |
| *sul1* | sulfamethoxazole | Sulfonamide | Target modification | CGGCGTGGGCTACCTGAACG | GCCGATCGCGTGAAGTTCCG | 433 | 60 | (14) |
| *dfrA12* | trimethoprim | Diaminopyrimidine | Target modification | CCTCTACCGAACCGTCACACA | GCGACAGCGTTGAAACAACTAC | 85 | 60 | (15) |
| *tetQ* | tetracycline | Tetracycline | Target protection | CGCCTCAGAAGTAAGTTCATACACTAAG | TCGTTCATGCGGATATTATCAGAAT | 108 | 60 | (15) |
| *qnrS1* | enrofloxacin | Fluoroquinolone | Target protection | GACGTGCTAACTTGCGTGAT | TGGCATTGTTGGAAACTTG | 118 | 63 | (16) |
| *intI1* | Not applicable | Not applicable | Not applicable | TGCCGTGATCGAAATCCAGATCCT | TTTCTGGAAGGCGAGCATCGTTTG | 109 | 60 | (17) |
| *intI2* | Not applicable | Not applicable | Not applicable | GTTATTTTATTGCTGGGATTAGGC | TTTTACGCTGCTGTATGGTGC | 164 | 56.5 | (18) |

### Table S7 Alpha diversity ± standard deviation for soil prokaryotic and fungal communities. Significance letters indicate significant differences determined with pairwise comparison determined by TukeyHSD when normally distributed and Kruskal-Wallis test followed by a Dunn test with fdr multiple testing correction if not normally distributed with antibiotic concentration and plant type as interaction term.*Significance of plant and treatment on fungal Shannon diversity was exceptionally determined with the ANOVA Welsch test followed by Howell’s test due to heteroscedasticity of the data.

| **Plant** | **Antibiotic treatment** | **Observed richness** | **Pielou’s evenness** | **Shannon diversity*** | **Inverse Simpson** |
| --- | --- | --- | --- | --- | --- |
| Prokaryotic community | | | | | |
| Radish | c0 | 18049 ± 376 ab | 2.007 ± 0.003 a | 8.54 ± 0.03 ab | 1436 ± 65 a |
|  | c0.1 | 17938 ± 766 ab | 2.004 ± 0.008 ab | 8.53 ± 0.07 abc | 1355 ± 153 ab |
|  | c1 | 17720 ± 366 b | 2.005 ± 0.003 ab | 8.52 ± 0.02 abc | 1381 ± 83 ab |
|  | c10 | 17573 ± 350 b | 1.998 ± 0.006 ab | 8.48 ± 0.03 bc | 1263 ± 95 ab |
| Spinach | c0 | 18165 ± 185 ab | 2.005 ± 0.003 ab | 8.54 ± 0.02 abc | 1356 ± 98 ab |
|  | c0.1 | 17949 ± 667 ab | 2.000 ± 0.011 ab | 8.51 ± 0.08 abc | 1314 ± 137 ab |
|  | c1 | 18434 ± 205 a | 2.006 ± 0.004 ab | 8.56 ± 0.02 a | 1417 ± 46 a |
|  | c10 | 17663 ± 401 b | 1.996 ± 0.005 b | 8.48 ± 0.02 c | 1231 ± 62 b |
| Fungal community | | | | | |
| Radish | c0 | 988.13 ± 39.60 a | 1.51 ± 0.11 a | 4.52 ± 0.35 a | 27.77 ± 15.32 a |
|  | c0.1 | 1020.78 ± 67.40 a | 1.58 ± 0.07 a | 4.77 ± 0.26 a | 34.33 ± 13.98 a |
|  | c1 | 1026.08 ± 41.28 a | 1.65 ± 0.03 a | 4.97 ± 0.12 a | 45.38 ± 6.37 a |
|  | c10 | 1050.29 ± 65.83 a | 1.61 ± 0.02 a | 4.88 ± 0.06 a | 39.88 ± 4.39 a |
| Spinach | c0 | 1025.63 ± 61.80 a | 1.63 ± 0.07 a | 4.89 ± 0.23 a | 42.36 ± 11.46 a |
|  | c0.1 | 1021.34 ± 50.29 a | 1.64 ± 0.07 a | 4.92 ± 0.23 a | 42.95 ± 11.70 a |
|  | c1 | 1048.87 ± 35.03 a | 1.62 ± 0.03 a | 4.90 ± 0.11 a | 41.67 ± 5.40 a |
|  | c10 | 1039.04 ± 38.46 a | 1.60 ± 0.04 a | 4.83 ± 0.15 a | 37.59 ± 9.83 a |

### Table S8 Prokaryotic and fungal community pairwise comparison results of the Bray-Curtis dissimilarity index based on PERMANOVA.

| Pairs | Df | SumsOfSqs | F.Model | R2 | p.value | p.adjusted | sig |
| --- | --- | --- | --- | --- | --- | --- | --- |
| Prokaryotic community | | | | | | | |
| c0 vs c10 | 1 | 0.234805824 | 5.468832104 | 0.199092269 | 0.001 | 0.006 | * |
| c0 vs c0.1 | 1 | 0.051389629 | 1.102509063 | 0.047722482 | 0.241 | 1 |  |
| c0 vs c1 | 1 | 0.114571563 | 2.752154056 | 0.111188467 | 0.001 | 0.006 | * |
| c10 vs c0.1 | 1 | 0.209991788 | 4.407396898 | 0.166900089 | 0.001 | 0.006 | * |
| c10 vs c1 | 1 | 0.142566828 | 3.34165317 | 0.131864056 | 0.001 | 0.006 | * |
| c0.1 vs c1 | 1 | 0.09356349 | 2.019073662 | 0.084061263 | 0.001 | 0.006 | * |
| Fungal community | | | | | | | |
| c0 vs c10 | 1 | 0.235313359 | 3.659335394 | 0.142612244 | 0.001 | 0.006 | * |
| c0 vs c0.1 | 1 | 0.058441409 | 0.842904707 | 0.036900067 | 0.637 | 1 |  |
| c0 vs c1 | 1 | 0.09010474 | 1.517499361 | 0.064526391 | 0.041 | 0.246 |  |
| c10 vs c0.1 | 1 | 0.207563173 | 3.252323682 | 0.128793046 | 0.001 | 0.006 | * |
| c10 vs c1 | 1 | 0.183534378 | 3.407383512 | 0.134109973 | 0.001 | 0.006 | * |
| c0.1 vs c1 | 1 | 0.061346953 | 1.041683268 | 0.045208645 | 0.343 | 1 |  |

### Table S9 Measured soil and plant parameters at the end of the experiment. Significance letters indicate significant differences determined with pairwise comparison determined by TukeyHSD when normally distributed and Kruskal-Wallis test followed by a Dunn test with fdr multiple testing correction if not normally distributed. ^1^nominal target concentration in mg kg^-1^ soil dry weight.

| Plant | Antibiotic treatment^1^ | Soil pH | Plant biomass (g) | Plant root biomass (g) | Plant leaves biomass (g) | Root C (g kg^-1^) | Root N (g kg^-1^) | Root C:N | Leaf C (g kg^-1^) | Leaf N (g kg^-1^) | Leaf C:N |
| --- | --- | --- | --- | --- | --- | --- | --- | --- | --- | --- | --- |
| Radish | c0 | 7.02 ± 0.06 a | 64.7 ± 6.79 a | 41.93 ± 5.23 a | 22.79 ± 1.81 a | 34.4 ± 1.62 b | 2.34 ± 0.30 a | 14.8 ± 1.68 a | 34.8 ± 1.16 a | 5.37 ± 0.45 a | 6.54 ± 0.75 ab |
|  | c0.1 | 6.95 ± 0.12 ab | 53.4 ± 10.1 ab | 35.28 ± 6.66 a | 18.08 ± 3.70 ab | 36.2 ± 0.82 a | 2.53 ± 0.25 a | 14.5 ± 1.64 a | 33.4 ± 1.30 a | 5.29 ± 0.36 a | 6.34 ± 0.53 b |
|  | c1 | 6.97 ± 0.12 ab | 48.7 ± 11.7 b | 31.61 ± 10.01 a | 17.12 ± 2.27 ab | 35.1 ± 0.28 ab | 2.63 ± 0.18 a | 13.4 ± 1.04 a | 33.4 ± 2.00 a | 5.41 ± 0.42 a | 6.20 ± 0.49 b |
|  | c10 | 6.86 ± 0.08 abc | 6.46 ± 7.78 c | 2.40 ± 3.18 b | 4.06 ± 4.74 c | 34.4 ± 0.13 b | 2.49 ± 0.20 a | 13.8 ± 1.05 a | 33.3 ± 2.21 a | 3.94 ± 0.74 b | 8.62 ± 1.49 a |
| Spinach | c0 | 6.71 ± 0.09 c | 16.7 ± 5.31 c | 0.36 ± 0.1 b | 14.50 ± 4.48 b | N.A. | N.A. | N.A. | 35.4 ± 0.90 a | 5.66 ± 0.20 a | 6.26 ± 0.13 b |
|  | c0.1 | 6.81 ± 0.08 bc | 15.7 ± 3.75 c | 0.37 ± 0.09 b | 13.72 ± 3.22 b | N.A. | N.A. | N.A. | 34.2 ± 0.73 a | 5.43 ± 0.23 a | 6.31 ± 0.30 b |
|  | c1 | 6.82 ± 0.16 abc | 14.7 ± 4.61 c | 0.31 ± 0.09 b | 12.83 ± 4.05 b | N.A. | N.A. | N.A. | 33.9 ± 0.93 a | 5.29 ± 0.09 a | 6.41 ± 0.12 b |
|  | c10 | 6.93 ± 0.12 ab | 15.8 ± 3.67 c | 0.32 ± 0.08 b | 14.01 ± 3.22 b | N.A. | N.A. | N.A. | 34.2 ± 1.09 a | 5.39 ± 0.38 a | 6.14 ± 0.24 b |

# Supplementary Figures

### Figure S1 PCoA Unconstrained analysis of prokaryotic (A) and fungal communities (B) with PERMANOVA


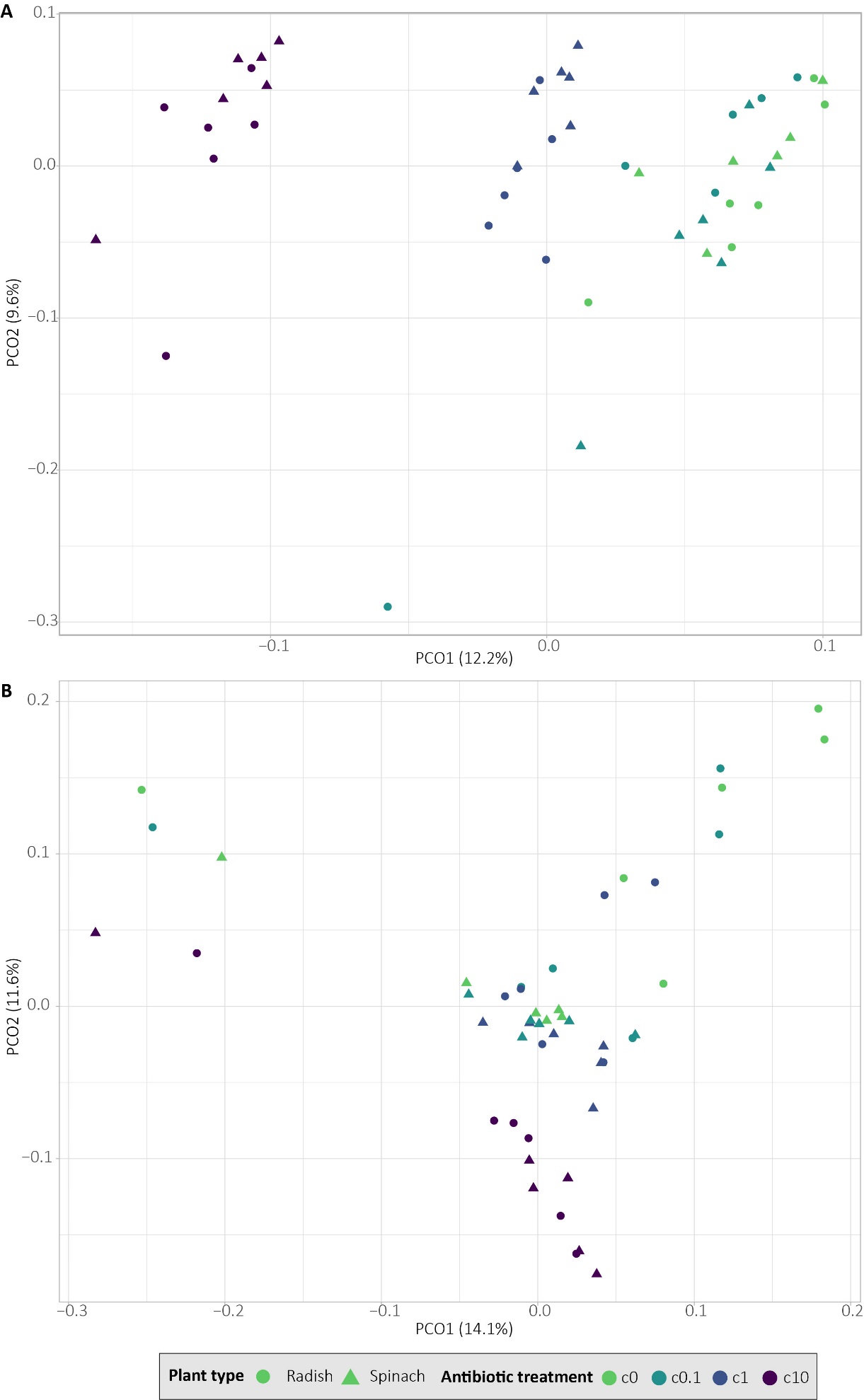


### Figure S2 Relative abundance of prokaryotic genera significantly impacted by antibiotic treatment and known for antibiotic-related mechanisms. Note that Janibacter contains both pollutant degrading and antibiotic resistance strains (Fig. 5).


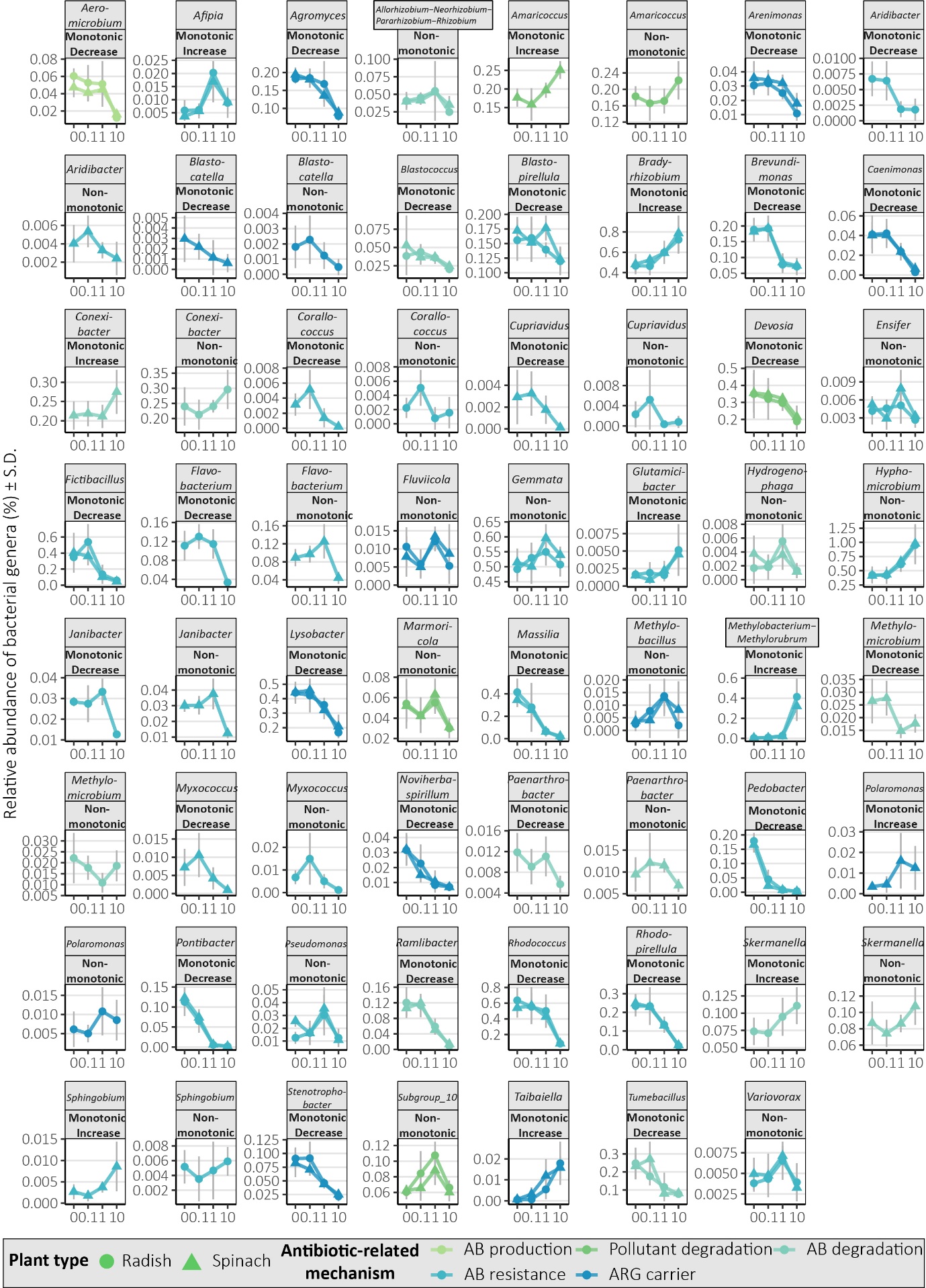


### Figure S3 In silico PCR results (<1000 bp for realistic PCR/qPCR results).


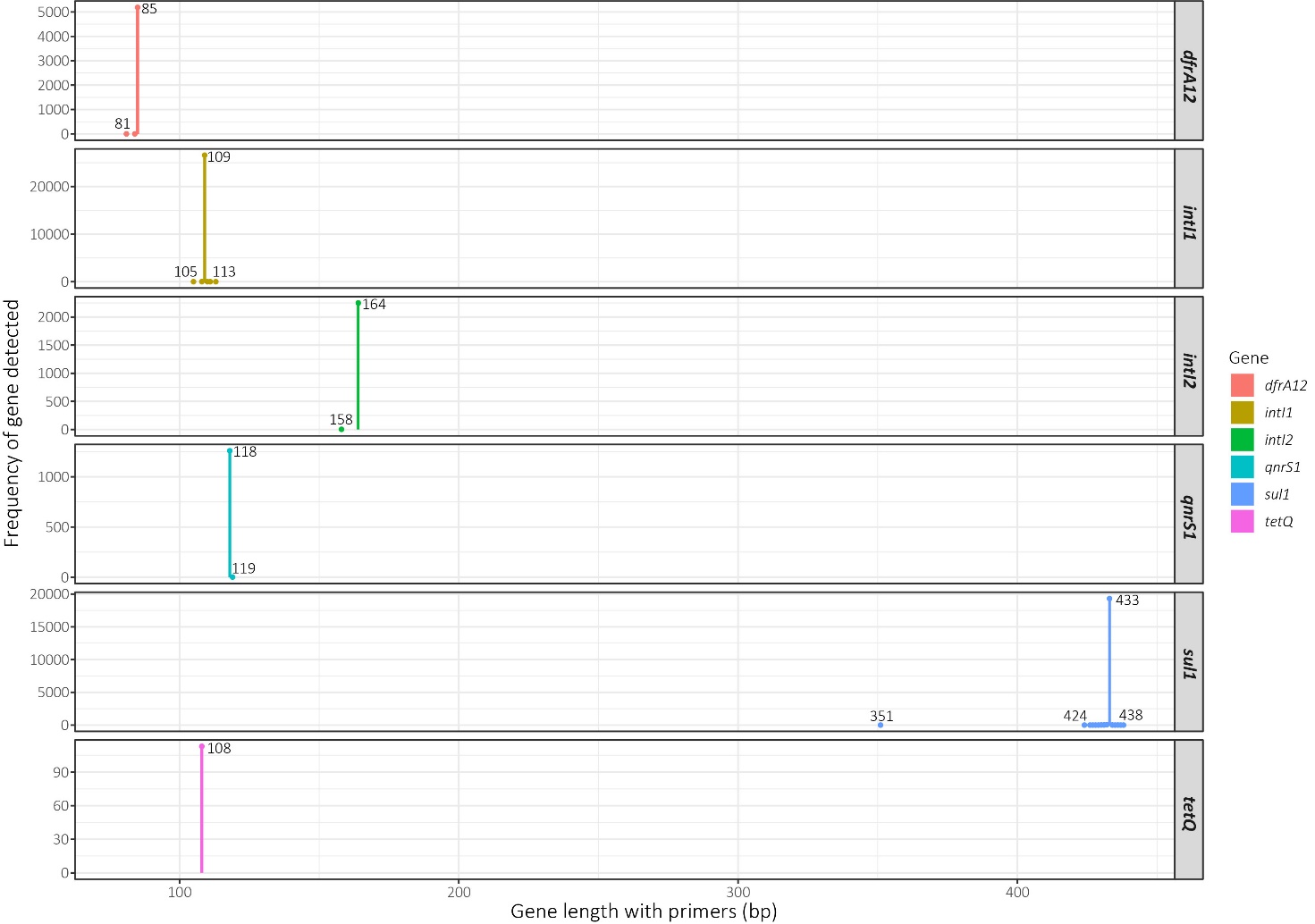


### Figure S4 Gene copies of intI1 and sul1 genes per 16S gene copy in g^-1^ dry soil as determined by qPCR. Differences in lowercase letters indicate significant differences of antibiotic treatments and plants for intI1, while differences in uppercase letters indicates significant differences of antibiotic treatments and plants for sul1 (p ≤ 0.05) as determined with pairwise comparison determined by Kruskal-Wallis followed by a Dunn test.


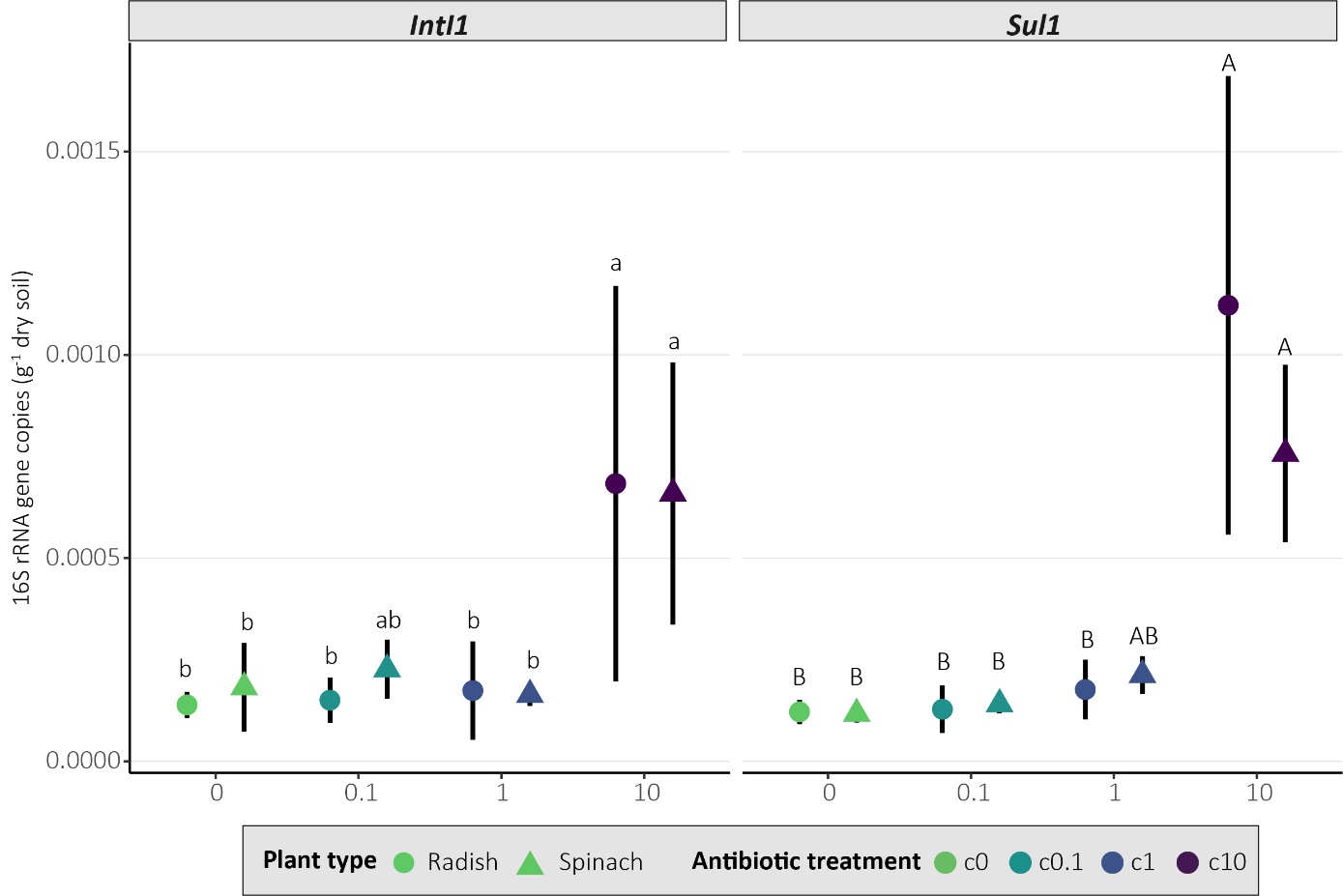


### Figure S5 Soil pH. Significance letters indicate significant differences determined with pairwise comparison determined by TukeyHSD.


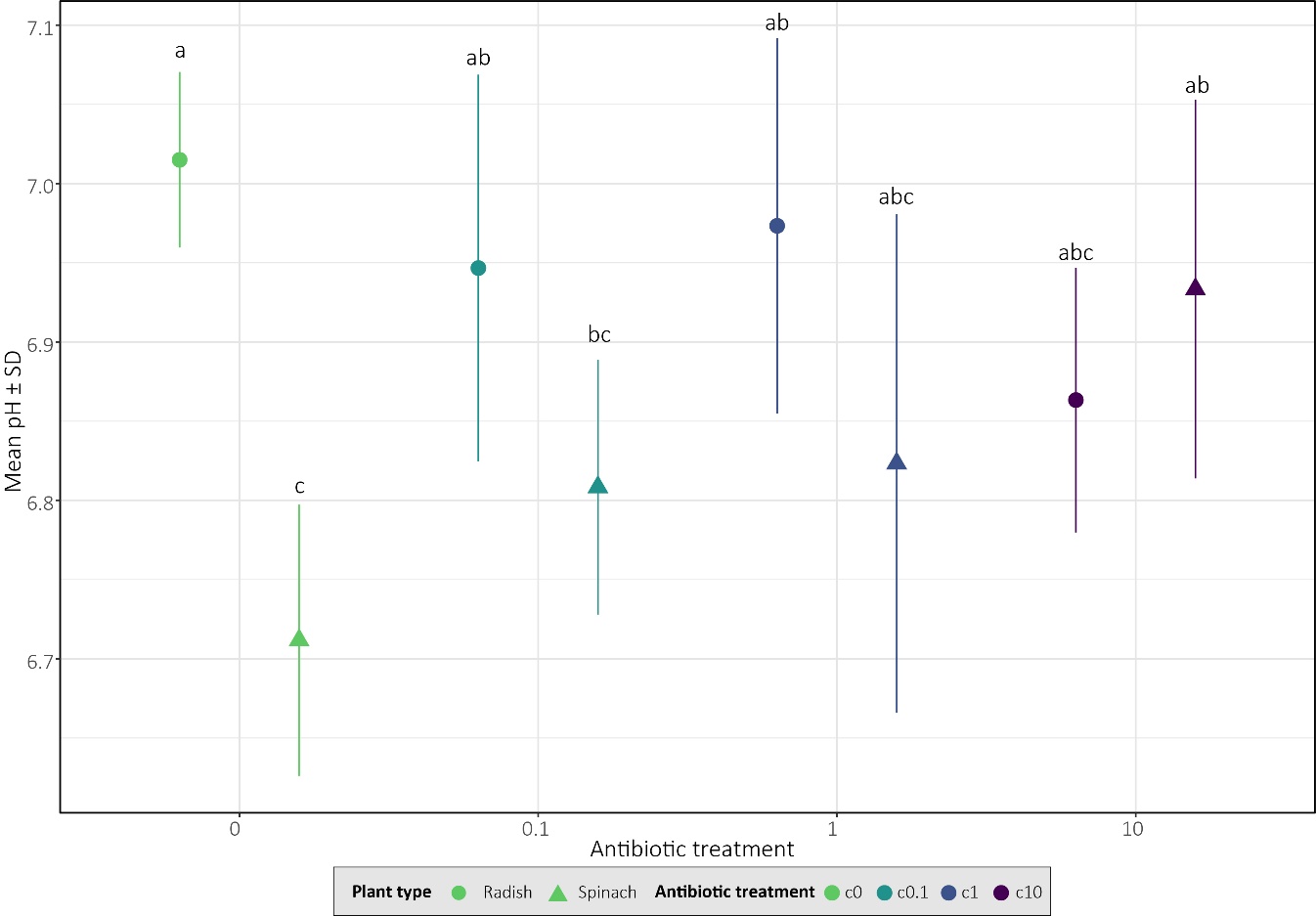


# References

1. Shi X, Zhang S, Zhang Y, Geng Y, Wang L, Peng Y, He Z. 2022. Novel and simple analytical method for simultaneous determination of sulfonamide, quinolone, tetracycline, macrolide, and chloramphenicol antibiotics in soil. Anal Bioanal Chem 414:6497–6506.

2. Thiele-Bruhn S. 2003. Pharmaceutical antibiotic compounds in soils - A review. J Plant Nutr Soil Sci 166:145–167.

3. Sarmah AK, Meyer MT, Boxall ABA. 2006. A global perspective on the use, sales, exposure pathways, occurrence, fate and effects of veterinary antibiotics (VAs) in the environment. Chemosphere 65:725–759.

4. Stephens R. 1956. Acidity Antibiotics 3:12–15.

5. Cycoń M, Mrozik A, Piotrowska-Seget Z. 2019. Antibiotics in the soil environment—degradation and their impact on microbial activity and diversity. Front Microbiol 10:1–45.

6. Nowara A, Burhenne J, Spiteller M. 1997. Binding of Fluoroquinolone Carboxylic Acid Derivatives to Clay Minerals. J Agric Food Chem 45:1459–1463.

7. Boxall ABA, Johnson P, Smith EJ, Sinclair CJ, Stutt E, Levy LS. 2006. Uptake of veterinary medicines from soils into plants. J Agric Food Chem 54:2288–2297.

8. McFarland JW, Berger CM, Froshauer SA, Hayashi SF, Hecker SJ, Jaynes BH, Jefson MR, Kamicker BJ, Lipinski CA, Lundy KM, Reese CP, Vu CB. 1997. Quantitative structure-activity relationships among macrolide antibacterial agents: In vitro and in vivo potency against Pasteurella multocida. J Med Chem 40:1340–1346.

9. Lin K, Gan J. 2011. Sorption and degradation of wastewater-associated non-steroidal anti-inflammatory drugs and antibiotics in soils. Chemosphere 83:240–246.

10. Avisar D, Primor O, Gozlan I, Mamane H. 2010. Sorption of sulfonamides and tetracyclines to montmorillonite clay. Water Air Soil Pollut 209:439–450.

11. Stoob K, Singer HP, Mueller SR, Schwarzenbach RP, Stamm CH. 2007. Dissipation and transport of veterinary sulfonamide antibiotics after manure application to grassland in a small catchment. Environ Sci Technol 41:7349–7355.

12. Frey B, Rime T, Phillips M, Stierli B, Hajdas I, Widmer F, Hartmann M. 2016. Microbial diversity in European alpine permafrost and active layers. FEMS Microbiol Ecol 92:1–17.

13. Tedersoo L, Lindahl B. 2016. Fungal identification biases in microbiome projects. Environ Microbiol Rep 8:774–779.

14. Kerrn MB, Klemmensen T, Frimodt-Möller N, Espersen F. 2002. Susceptibility of Danish Escherichia coli strains isolated from urinary tract infections and bacteraemia, and distribution of sul genes conferring sulphonamide resistance. J Antimicrob Chemother 50:513–516.

15. Hu HW, Wang JT, Li J, Li JJ, Ma YB, Chen D, He JZ. 2016. Field-based evidence for copper contamination induced changes of antibiotic resistance in agricultural soils. Environ Microbiol 18:3896–3909.

16. Marti E, Balcázar JL. 2013. Real-time PCR assays for quantification of qnr genes in environmental water samples and chicken feces. Appl Environ Microbiol 79:1743–1745.

17. Rosewarne CP, Pettigrove V, Stokes HW, Parsons YM. 2010. Class 1 integrons in benthic bacterial communities: Abundance, association with Tn402-like transposition modules and evidence for coselection with heavy-metal resistance. FEMS Microbiol Ecol 72:35–46.

18. Luo Y, Mao D, Rysz M, Zhou Q, Zhang H, Xu L, Alvarez PJJ. 2010. Trends in antibiotic resistance genes occurrence in the Haihe River, China. Environ Sci Technol 44:7220–7225.
